# EGFR-mediated activation of adipose tissue macrophages promotes obesity and insulin resistance

**DOI:** 10.1038/s41467-022-32348-3

**Published:** 2022-08-10

**Authors:** Shirong Cao, Yu Pan, Jiaqi Tang, Andrew S. Terker, Juan Pablo Arroyo Ornelas, Guan-nan Jin, Yinqiu Wang, Aolei Niu, Xiaofeng Fan, Suwan Wang, Raymond C. Harris, Ming-Zhi Zhang

**Affiliations:** 1 Division of Nephrology and Hypertension, Department of Medicine, Vanderbilt University Medical Center, Nashville, TN USA; 2 Vanderbilt Center for Kidney Disease, Vanderbilt University Medical Center, Nashville, TN USA; 3 Division of Nephrology, Shanghai Ninth People’s Hospital, Shanghai Jiao Tong University School of Medicine, Shanghai, China; 4 Veterans Affairs, Nashville, TN USA

**Keywords:** Metabolic syndrome, Obesity, Innate immunity

## Abstract

Obesity and obesity-related health complications are increasing in prevalence. Adipose tissue from obese subjects has low-grade, chronic inflammation, leading to insulin resistance. Adipose tissue macrophages (ATMs) are a source of proinflammatory cytokines that further aggravate adipocyte dysfunction. In response to a high fat diet (HFD), ATM numbers initially increase by proliferation of resident macrophages, but subsequent increases also result from infiltration in response to chemotactic signals from inflamed adipose tissue. To elucidate the underlying mechanisms regulating the increases in ATMs and their proinflammatory phenotype, we investigated the role of activation of ATM epidermal growth factor receptor (EGFR). A high fat diet increased expression of EGFR and its ligand amphiregulin in ATMs. Selective deletion of EGFR in ATMs inhibited both resident ATM proliferation and monocyte infiltration into adipose tissue and decreased obesity and development of insulin resistance. Therefore, ATM EGFR activation plays an important role in adipose tissue dysfunction.

## Abstract (web summary)

In obesity, adipose tissue is chronically inflamed, leading to insulin resistance, and adipose tissue macrophages (ATMs) are a major source of cytokines that aggravate adipocyte dysfunction. Here, the authors show that selective deletion of the epidermal growth factor receptor in ATMs decreased obesity and development of insulin resistance.

## Introduction

There is increasing prevalence of obesity and obesity-related health complications throughout the world. The increased adipose tissue of obesity is associated with a state of low-grade, chronic inflammation and dysregulated metabolism, leading to insulin resistance and development of metabolic syndrome. It is recognized that adipose tissue from obese individuals and experimental animals has increased macrophages (ATMs) that are a source of proinflammatory cytokines that further aggravate adipocyte dysfunction. ATM number can increase in response to obesogenic stimuli such as a high fat diet (HFD). ATM number initially increases through proliferation of resident macrophages, but subsequent increases can result from infiltration in response to chemotactic signals from inflamed adipose tissue^1–4^. However, the underlying mechanisms regulating the increases in ATMs and their proinflammatory phenotype have not been completely elucidated.

The epidermal growth factor receptor (EGFR) is a member of the family of ErbB receptors, which consists of an extracellular ligand-binding domain, a single membrane-spanning region, a homologic cytoplasmic protein tyrosine kinase domain and a C-terminal tail with multiple phosphorylation sites^5^. Ligand binding to EGFR leads to activation of the intrinsic kinase domain and subsequent phosphorylation on specific tyrosine residues within the cytoplasmic tail. These phosphorylated residues serve as docking sites for a variety of signaling molecules whose recruitment leads to the activation of intracellular pathways controlling cell proliferation, differentiation, and apoptosis^5, 6^. EGFR can be activated by a family of ligands including EGF, TGF-α, HB-EGF, amphiregulin, epiregulin and betacellulin^7^. We previously found that an EGFR tyrosine kinase inhibitor decreased obesity in a murine model of leptin receptor deficient type II diabetes^8^. In a lung cancer patient with diabetes, treatment with an EGFR tyrosine kinase inhibitor led to improved glucose control^9^.

EGFR has been reported to be expressed in many tissues and cells, including cultured 3T3-L1 adipocytes^10^, skeletal muscle^11^, and hepatocytes^12^. Macrophages also express EGFR. Previous studies indicated that selective deletion of myeloid EGFR reduced inflammation in an experimental model of colitis^13^. However, the role of EGFR in these cell types in obesity has not been previously investigated. In the current studies we confirmed that mice with an intrinsic global EGFR tyrosine kinase defect had less obesity and metabolic dysfunction in response to a high fat diet. However, selective deletion of EGFR in peripheral insulin-sensitive tissues, hepatocytes, skeletal muscle or adipocytes, failed to alter the onset of obesity and the insulin resistance. In contrast, both EGFR and its ligand, amphiregulin, markedly increased in ATMs in HFD-induced obesity, and selective deletion of EGFR in ATMs reduced HFD-induced obesity, adipose tissue derangements and insulin resistance. Deletion of EGFR in ATMs inhibited both resident ATM proliferation and monocyte recruitment into adipose tissue. Therefore, these studies demonstrated that EGFR activation in ATMs plays an important role to mediate adipose tissue dysfunction.

## Results

### EGFR tyrosine kinase deficiency protected against high fat diet-induced insulin resistance

**Fig. 1:**
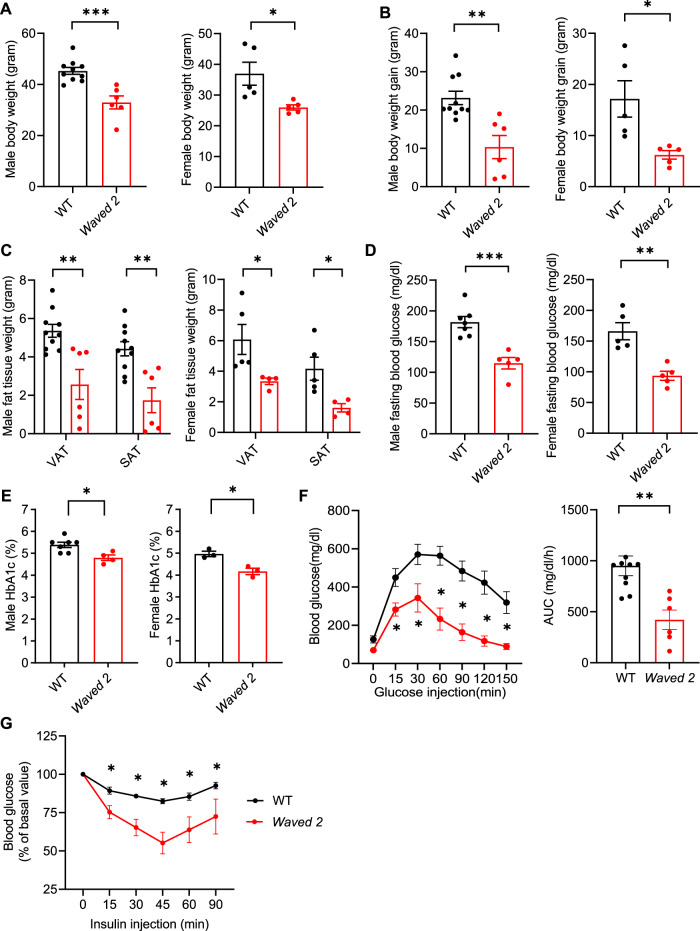
*Waved* 2 mice developed less metabolic derangement in diet induced obesity. *Waved* 2 mice with intrinsic EGFR tyrosine kinase deficiency and WT mice were fed the HFD for 12 weeks. **A**, **B** Both male and female *Waved 2* mice had lower body weight (Male: *n* = 6 and 10; female: *n* = 5) **A** and body weight gain (Male: *n* = 6 and 10; female: *n* = 5) **B**. **C** Both male and female *Waved 2* mice had less visceral adipose tissue (VAT) and subcutaneous adipose tissue (SAT) mass compared to WT mice (Male: *n* = 6 and 10; female: *n* = 4 and 5). **D**, **E** Both male and female *Waved 2* mice had lower fasting blood glucose (Male: *n* = 5 and 7; female: *n* = 5) **D** and HbA1c (Male: n = 5 and 7; female: *n* = 3) **E**. *N* = 5 and 7. **F**, **G** Male *Waved 2* mice had improved glucose tolerance (*n* = 6 and 9) **F** and insulin tolerance (*n* = 4) **G**. Data are means ± SEM, *P < 0.05, **P < 0.01, ***P < 0.001, analyzed using 2 tailed Student’s t test for **A**, **B**, **D**, and **E**; 2-way ANOVA followed by Bonferroni’s post hoc test for **C**; 2 tailed Student’s t test and 2-way ANOVA followed by Tukey’s post hoc test for **F**; and 2-way ANOVA followed by Tukey’s post hoc test for **G**.

To investigate the potential role of EGFR activation in the development of insulin resistance caused by the HFD, we first studied *Waved 2* mice, which have a point mutation in EGFR that reduces intrinsic tyrosine kinase activity by >90% globally^8^. We recently reported that differential renal EGFR expression plays a role in the sex difference associated susceptibility to progressive kidney injury^14^. Therefore, both male and female *Waved 2* mice were fed the HFD for up to 12 weeks. Both male and female *Waved* 2 mice had decreased relative body weights and absolute increases in body weight gain and less increase in subcutaneous fat tissue (SAT) and visceral fat tissue (VAT) compared to their littermate wild type (WT) mice (Fig. 1A–C). Additionally, after 12 weeks on the HFD, *Waved 2* mice had lower fasting blood glucose and HbA1c levels (Fig. 1D, E), and improved glucose tolerance tests and insulin tolerance tests compared to WT mice (Fig. 1F, G and Supplementary Fig. 1).

### EGFR deletion in peripheral insulin sensitive tissues did not prevent development of high fat diet-induced insulin resistance

To investigate whether EGFR in hepatocytes mediated insulin resistance resulting from the HFD, we generated mice with selective EGFR deletion in hepatocytes (Alb1-Cre; EGFR^f/f^, hepatocyte EGFR^−/−^) and corresponding WT (EGFR^f/f^) mice and fed them the HFD for 12 weeks. EGFR deletion in hepatocytes was confirmed by reduced liver mRNA levels in hepatocyte EGFR^−/−^ mice (Fig. S2A). Body weight, fasting blood glucose, and HbA1c were comparable in hepatocyte EGFR^−/−^ mice and WT mice after the HFD treatment (Supplementary Fig. 2B–D). Similar insulin sensitivity was observed in both liver and fat tissue in hepatocyte EGFR^−/−^ and WT mice, as indicated by comparable acute insulin-stimulated Akt phosphorylation (data not shown).

We also generated mice with selective EGFR deletion in skeletal muscle (ACTA1-Cre; EGFR^f/f^, smEGFR^−/−^) and WT (EGFR^f/f^) mice and fed them the HFD for 12 weeks. Both decreased EGFR immunofluorescence and *Egfr* mRNA levels confirmed effective EGFR deletion in vastus lateralis in smEGFR^−/−^ mice (Supplementary Fig. 3A). Body weight, fasting blood glucose, HbA1c, glucose tolerance tests, and insulin tolerance tests were again comparable in smEGFR^−/−^ mice and WT mice (Supplementary Fig. 3B–F).

To determine whether the effects seen in *Waved 2* mice were mediated by inhibition of adipocyte EGFR signaling, we generated mice with selective EGFR deletion in adipocytes (Adipoq-Cre; EGFR^f/f^, Adipocyte EGFR^−/−^) and WT (EGFR^f/f^) mice and fed them the HFD for 16 weeks. Efficient EGFR deletion in adipose tissue was confirmed by decreased EGFR mRNA expression and EGFR immunofluorescence in epididymal fat tissue (Supplementary Fig. 4A, B). Body weight, fasting blood glucose, and HbA1c were comparable in male Adipocyte EGFR^−/−^ mice and WT mice throughout the HFD treatment (Supplementary Fig. 4C–E). Adipocyte EGFR^−/−^ mice and WT mice also had similar glucose tolerance tests and insulin tolerance tests (Supplementary Fig. 4F, G). Body weight, fasting blood glucose and HbA1c were also comparable in female Adipocyte EGFR^−/−^ mice and WT mice in response to the HFD (Supplementary Fig. 5).

These negative results indicated that the protection against insulin resistance seen in *Waved 2* mice in response to the HFD was not due to deficient EGFR activity in the primary peripheral insulin sensitive cell tissues.

### Both EGFR and its ligand, amphiregulin, were selectively upregulated in adipose tissue macrophage (ATMs) in response to the HFD

**Fig. 2:**
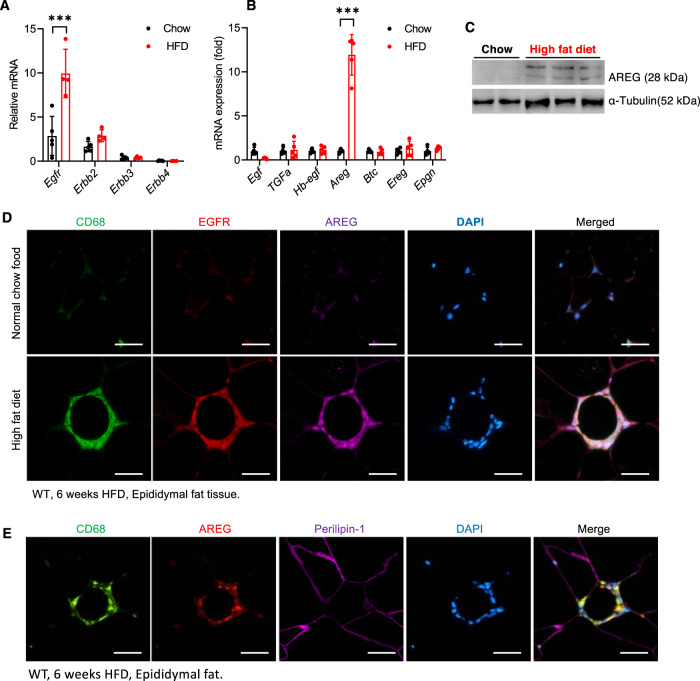
Both EGFR and amphiregulin expression were selectively increased in ATMs in WT mice 6 weeks after the HFD. **A** HFD treatment led to selective increases in *Egfr* mRNA levels in epididymal fat tissue. *N* = 4 and 5. **B** HFD treatment also led to selective increases in EGFR ligand, *Areg* mRNA levels in epididymal fat tissue. *N* = 4 and 5. **C** Immunoblotting confirmed increased EF AREG in HFD-treated mice. **D** Representative photomicrographs of 3 independent experiments showed that both EGFR and AREG expression was minimal in mice with chow food but was evident and primarily colocalized with CD68 in ATMs. Scale bar = 100 μm. **E** In HFD-treated mice, AREG expression in EF adipocytes was minimal (representative of 3 independent experiments). Scale bar = 100 μm. Data are means ± SEM, ***P < 0.001, analyzed using 2-way ANOVA followed by Bonferroni’s post hoc test for **A** and **B**.

Among the four members of the *ErbB* family, only *Erbb1* (*Egfr*) mRNA expression was increased in visceral epididymal fat (EF) in mice fed the HFD for 6 weeks (Fig. 2A). Furthermore, among all EGFR ligands, only *Areg* (amphiregulin) mRNA expression was markedly increased (>10 times) in EF in response to the HFD (Fig. 2B), which was confirmed by immunoblotting (Fig. 2C). There was minimal detectable EGFR or AREG by immunostaining in EF in mice fed chow food, but expression of both receptor and ligand was clearly evident in mice on the HFD for 6 weeks (Fig. 2D). Increased expression of both EGFR and AREG was restricted and colocalized with CD68, a marker of ATMs, in the crown-like structure (CLSs) in EF (Fig. 2D). In contrast, there was minimal AREG expression colocalization with perilipin, indicating relatively less expression in adipocytes (Fig. 2E). Similar to *Waved* 2 mice, global AREG^−/−^ mice were protected against insulin resistance in response to the HFD, as indicated by lower body weight and fasting blood glucose as well as improved glucose tolerance tests and insulin tolerance tests compared to WT mice (Supplementary Fig. 6).

### Mice with selective EGFR deletion in myeloid cells selectively attenuated white adipose tissue insulin resistance caused by the HFD

**Fig. 3:**
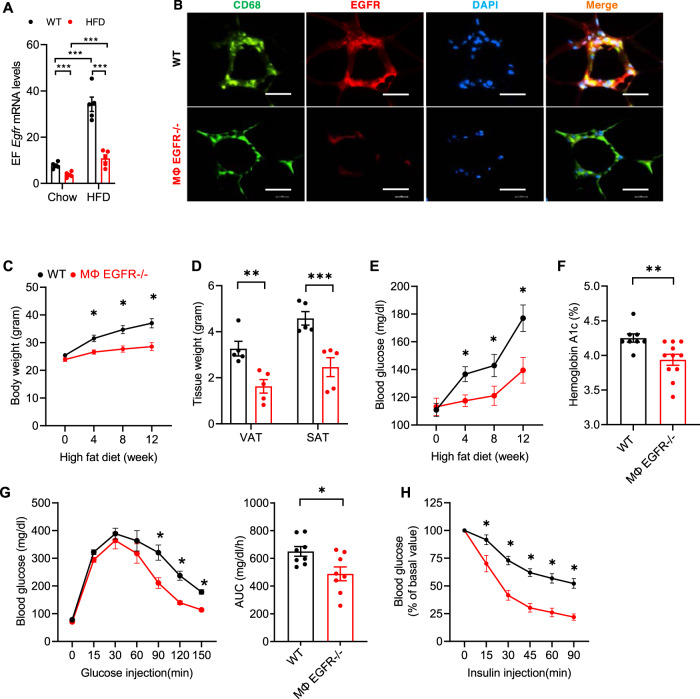
Myeloid EGFR deficiency attenuated insulin resistance in diet-induced obesity. WT (EGFR^f/f^) mice and MΦ EGFR^−/−^ (CD11b-Cre; EGFR^f/f^) mice were fed the HFD for 12 weeks. **A** Epididymal fat (EF) *Egfr* mRFNA levels were markedly increased in WT mice response to the HFD. MΦ EGFR^−/−^ mice had markedly lower EF *Egfr* mRNA levels compared to WT mice with chow food or with the HFD. *N* = 5. **B** Representative images showed that EGFR immunofluorescence was minimal in MΦ EGFR^−/−^ mice but clearly evident in ATMs in WT mice. Scale bar = 100 μm. **C**–**F** MΦ EGFR^−/−^ mice had reduced increases in body weight (*n* = 10 and 11) **C**, visceral and subcutaneous fat tissue (VAT and SAT) (*n* = 5) **D** fasting blood glucose (*n* = 9 and 11) **E** and HbA1c (*n* = 8 and 11) **F**. **G**, **H** MΦ EGFR^−/−^ mice exhibited improved glucose tolerance (*n* = 8 and 10) **G** and insulin tolerance (*n* = 9) **H**. Data are means ± SEM, *P < 0.05, **P < 0.01, ***P < 0.001, analyzed using 2 tailed Student’s t test for **F**; 2-way ANOVA followed by Tukey’s post hoc test for **C**, **E**, and **H**; 2-way ANOVA followed by Bonferroni’s post hoc test for **A** and **D**; and 2 tailed Student’s t test and 2-way ANOVA followed by Tukey’s post hoc test for **G**.

We generated mice with selective myeloid EGFR deletion (CD11b-Cre; EGFR^f/f^, MΦ EGFR^−/−^). MΦ EGFR^−/−^ mice exhibited no differences in bone marrow or peripheral monocyte levels compared to corresponding WT mice (Supplementary Fig. 7). *Egfr* mRNA levels were markedly reduced in epididymal adipose tissue in MΦ EGFR^−/−^ mice that were on chow food or the HFD for 12 weeks (Fig. 3A). Immunofluorescent staining confirmed markedly decreased EGFR in ATMs in CLSs in MΦ EGFR^−/−^ mice (Fig. 3B). MΦ EGFR^−/−^ mice had significantly less body weight gain compared to WT mice, beginning as early as 4 weeks after initiation of the HFD (Fig. 3C). Both VAT mass and SAT mass were significantly lower in MΦ EGFR^−/−^ mice than WT mice after 12 weeks on the HFD (Fig. 3D). MΦ EGFR^−/−^ mice also had significantly lower fasting blood glucose compared to WT mice, beginning as early as 4 weeks after the HFD (Fig. 3E). MΦ EGFR^−/−^ mice had lower HbA1c and improved glucose tolerance tests and insulin tolerance tests after 12 weeks on the HFD (Fig. 3F–H).

**Fig. 4:**
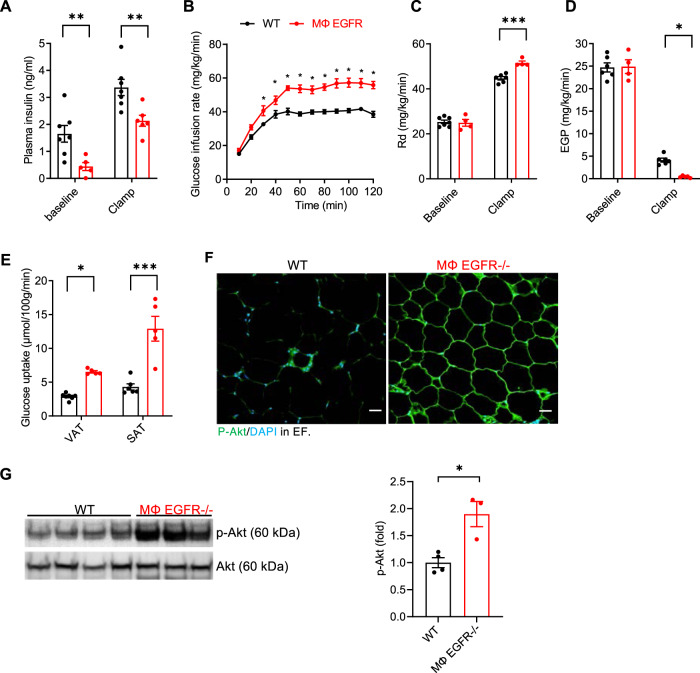
Mice with selective EGFR deletion in myeloid cells selectively attenuated white adipose tissue insulin resistance in diet-induced obesity. Hyperinsulinemic-euglycemic clamps were performed on 5 h fasted WT and MΦ EGFR^−/−^ mice after 12 weeks on the HFD. **A** MΦ EGFR^−/−^ mice had lower plasma insulin levels at baseline and during clamp periods. *N* = 5 and 7. **B** MΦ EGFR^−/−^ mice had less severe insulin resistance, as more glucose infusion was needed to maintain a constant blood glucose. *N* = 5 and 7. **C**, **D**. MΦ EGFR^−/−^ mice had increased rates of glucose disappearance (Rd) (*N* = 4 and 7) **C** and decreased endogenous glucose production (EGP) (*N* = 4 and 7) **D**. **E** MΦ EGFR^−/−^ mice had increased glucose uptake, a marker of insulin resistance in VAT and SA. N = 5 and 7. **F** Representative images showed more insulin-stimulated p-Akt in EF in MΦ EGFR^−/−^ mice. Scale bar = 100 μm. **G** Immunoblotting determined higher insulin-stimulated p-Akt in EF in MΦ EGFR^−/−^ mice. *N* = 3 and 4). Data are means ± SEM, *P < 0.05, **P < 0.01, ***P < 0.001, analyzed using 2-way ANOVA followed by Bonferroni’s post hoc test for **A**, and **C**–**E**; 2-way ANOVA followed by Tukey’s post hoc test for **B**; and 2 tailed Student’s t test for **G**.

The hyperinsulinemic-euglycemic clamp, or insulin clamp, is a “gold standard” method for assessing insulin action in vivo^15^. During an insulin clamp, hyperinsulinemia is achieved by a constant insulin infusion. Euglycemia is maintained via the variable glucose infusion rate (GIR), which is determined by measuring blood glucose at brief intervals throughout the experiment and is indicative of whole-body insulin action, as mice with enhanced insulin action require a greater GIR. We performed insulin clamps on 5 h fasted mice after 12 weeks on the HFD. MΦ EGFR^−/−^ mice had lower insulin levels at baseline and after 5 h fasting (Fig. 4A) and required administration of significantly more glucose in order to maintain a constant blood glucose throughout the course of the study, an indication of less insulin resistance (Fig. 4B). MΦ EGFR^−/−^ mice also exhibited an increased rate of glucose disappearance (Rd) (Fig. 4C), consistent with lower insulin concentrations needed for tissue glucose utilization as well as lower hepatic endogenous glucose production (EGP) (Fig. 4D). MΦ EGFR^−/−^ mice had significant increases in glucose uptake into SAT and VAT, but not into brown-AT, skeletal muscle, heart or brain, an indication of a white adipose tissue-specific effect (Fig. 4E and Supplementary Fig. 8). Immunofluorescence and immunoblotting confirmed increased insulin-stimulated p-AKT expression in epididymal fat in MΦ EGFR^−/−^ mice (Fig. 4F, G). AKT is a downstream signaling of insulin signaling in adipocytes, and increased activation of ATK (p-AKT) is an indication of greater insulin sensitivity, although AKT can also be activated via other mechanisms such as insulin growth factor 1^16^.

We assessed energy balance by indirect calorimetry experiments using a Promethion metabolic cage system in MΦ EGFR^−/−^ mice and littermate WT controls with chow food and with HFD for 4 weeks through Vanderbilt MMPC^17^. Body composition was determined by NMR (Bruker Minispec). MΦ EGFR^−/−^ mice fed the HFD had reduced increases in fat mass, which contributed to the reduced increases in body weight (Supplementary Fig. 9). However, MΦ EGFR^−/−^ mice and littermate controls showed comparable food ingestion with similar circadian rhythms (Supplementary Figs. 10 and 11). Indirect calorimetry measurements including respiratory exchange ratio (RER), oxygen consumption, carbon dioxide production, energy expenditure and energy balance were comparable between WT and KO groups although there was a trend for increased ambulatory/pedestrian locomotion, locomotor activity, and distance traveled in the cage by the MΦ EGFR^−/−^ mice (Supplementary Figs. 10 and 11).

### Myeloid EGFR^−/−^ mice had an increased number of smaller adipocytes and less adipose tissue fibrosis in response to the HFD

**Fig. 5:**
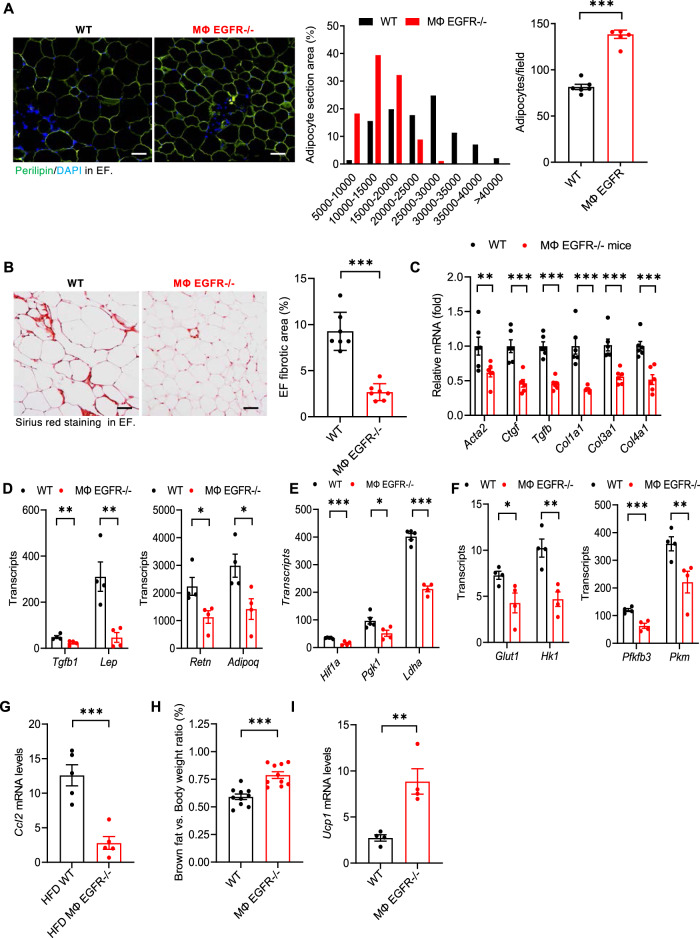
Myeloid EGFR^−/−^ mice had reduced adipocyte hypertrophy and adipose tissue adipokines, glycolysis, hypoxia, and fibrosis in response to the HFD. WT and MΦ EGFR^−/−^ mice were fed the HFD for 12 weeks. **A** Image analysis of perilipin immunofluorescence showed smaller but increased adipocytes in EF in MΦ EGFR^−/−^ mice than WT mice. *N* = 10, scale bar = 100 μm. **B** Quantitative Sirius Red staining indicated less fibrosis in EF in MΦ EGFR^−/−^ mice than WT mice. *N* = 6, scale bar = 100 μm. **C**–**G** MΦ EGFR^−/−^ mouse EF had lower profibrotic and fibrotic genes including *Acta2*, *Ctgf*, *Tgfb1*, *Col1a1*, *Col3a1*, and *Col4a1*, *n* = 6 **C**, lower adipokine genes including *Lep*, *Adipoq*, *Retn*, and *Tgfb1*, *n* = 4 **D**, lower hypoxia-related genes including *Hif1a*, *Pgk1*, and *Ldha*, *n* = 4 **E**, lower glycolytic genes including *Glut1, Hk1, Pfkfb3, and Pkm*, *n* = 4 **F**, and lower chemoattractant *Ccl2* gene, *n* = 5 **G**. **H**, **I** MΦ EGFR^−/−^ mice had higher interscapular brown fat, *n* = 10 **H** and higher inguinal fat *Ucp1* transcripts, a beige marker, *n* = 4 **I**. Data are means ± SEM, **P < 0.01, ***P < 0.001, analyzed using 2 tailed Student’s *t* test for **A**, **B**, **G**–**I**, and 2-way ANOVA followed by Bonferroni’s post hoc test for **C**–**F**.

Both increased adipocyte size (hypertrophy) and inappropriate extracellular matrix remodeling (fibrosis) contribute to the pathogenesis of dysfunctional adipose tissue in obesity^18, 19^. Image analysis of perilipin immunofluorescence demonstrated that mean adipocyte size was significantly smaller in MΦ EGFR^−/−^ epididymal fat, with a shift to an increased number of smaller adipocytes and increased adipocyte density (Fig. 5A). Sirius red staining demonstrated less fibrosis in epididymal fat tissue in MΦ EGFR^−/−^ mice (% of fibrosis area: 2.67 ± 0.34 vs. 9.27 ± 0.78 of WT mice, *P* < 0.001, *n* = 7) (Fig. 5B). Compared to WT mice, epididymal fat in MΦ EGFR^−/−^ mice had lower mRNA levels of profibrotic and fibrotic components, including *Acta2*, *Ctgf*, *Tgfb*, *Col1a1*, *Col3a1*, and *Col4a1* (Fig. 5C). Compared to WT mice, MΦ EGFR^−/−^ epididymal fat also had lower mRNA levels of adipokine genes including *Lep*, *Adipoq*, *Retn*, and *Tgfb*, (Fig. 5D), hypoxia genes including *Hif1a*, and *Pgk1* and *Ldha* (Fig. 5E) and glycolytic genes including *Glut1*, *Hk1*, *Pfkfb3*, and *Pkm* (Fig. 5F) and *Ccl2* (Fig. 5G).

Brown adipose tissue (BAT) regulates body temperature through non-shivering thermogenesis to dissipate chemical energy to produce heat. Beige adipocytes are interspersed within the white adipose tissue (particular in SAT) in rodents, and white adipose tissue can undergo a process known as browning, indicated by increased beige adipocytes. Brown and activated beige cells are characterized by high levels of fatty acid β-oxidation, mitochondrial content, and thermogenesis^20^. We determined that after 12 weeks on the high fat diet, MΦ EGFR^−/−^ mice had relatively higher BAT mass and inguinal fat tissue *Ucp1* mRNA levels compared to WT mice (Fig. 5H, I).

### Myeloid EGFR^−/−^ adipose tissue had less ATM accumulation with less inflammation

**Fig. 6:**
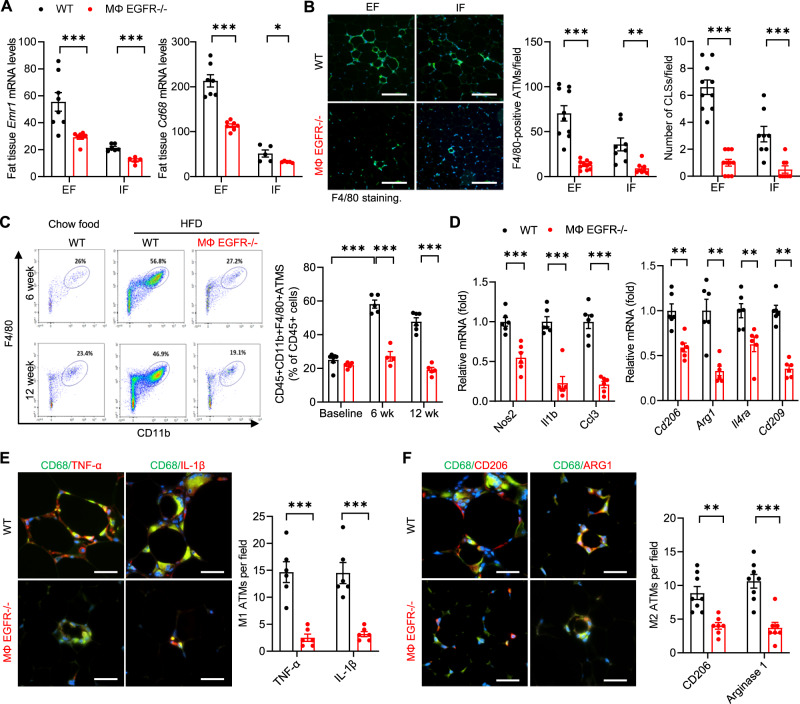
Myeloid EGFR^−/−^ mice had less ATM accumulation and lower proinflammatory cytokines/chemokine in diet-induced obesity. WT and MΦ EGFR^−/−^ mice were fed the HFD for 6 and 12 weeks. **A** MΦ EGFR^−/−^ mice had lower *Emr1* (F4/80) (*N* = 5 and 8) and *Cd68* (*N* = 5 and 8) expression in both EF and IF compared to WT mice at 12 weeks after the HFD. **B** Quantitative F4/80 staining demonstrated fewer ATMs and CLSs in both EF and IF in MΦ EGFR^−/−^ mice than WT mice at 12 weeks after the HFD. *N* = 8 and 10, scale bar = 200 μm. **C** Flow cytometry confirmed less EF CD45^+^CD11b^+^F4/80^+^ ATMs at both 6 and 12 weeks after the HFD in MΦ EGFR^−/−^ mice than WT mice. *N* = 4–6. **D** MΦ EGFR^−/−^ mice had lower EF mRNA expression of both proinflammatory cytokines (*Nos2*, *Il1b*, and *Ccl3*) and antiinflammatory cytokines (*Cd206*/*Mrc1*, *Arg1*, *Il4ra*, and *Cd209*) at 12 weeks on the HFD. *N* = 6. **E**, **F** Double immunofluorescent staining demonstrated fewer M1 ATMs (CD68^+^TNF^+^ and CD68^+^IL-1β^+^ positive cells) (*N* = 6) and M2 ATMs (CD68^+^CD206^+^ and CD68^+^Arg1^+^ positive cells) (*N* = 8) in EF in MΦ EGFR^−/−^ mice than WT mice. Scale bar = 100 μm. Data are means ± SEM, **P < 0.01, ***P < 0.001, analyzed using 2-way ANOVA followed by Bonferroni’s post hoc test for all.

In obesity, the increased ATMs are primarily located around dead adipocytes in the CLSs, which clear dead or dying adipocytes and buffer lipids. CLSs are considered a histologic hallmark of increased adipose tissue proinflammatory cytokines and insulin resistance^21^. In response to the HFD, mRNA levels of both *Emr1* (F4/80) and *Cd68*, two markers of ATMs, were significantly higher in EF than in inguinal fat (IF), indicating more ATM accumulation in visceral fat than in subcutaneous fat. *Emr1* and *Cd68* mRNA levels were markedly reduced in both visceral fat and subcutaneous fat in MΦ EGFR^−/−^ mice compared to WT mice in response to the HFD (Fig. 6A). F4/80 and CD68 immunofluorescent staining indicated increased ATMs and CLSs in EF compared to IF in WT mice and significantly fewer ATMs and CLSs in both EF and IF in MΦ EGFR^−/−^ mice compared to WT mice (Fig. 6B and Supplementary Fig. 12). Flow cytometry confirmed markedly fewer ATMs (F4/80^+^ CD11b^+^) in EF from MΦ EGFR^−/−^ mice compared to WT mice at both 6 weeks and 12 weeks after initiation of the HFD (Supplementary Fig. 13 and Fig. 6C). The mRNA levels of both the proinflammatory M1 cytokines (*Nos2*, *Il1b*, and *Ccl3*) and the antiinflammatory M2 cytokines (*Cd206*/*Mrc1*, *Arg1*, *Il4ra*, and *Cd209*) were markedly lower in MΦ EGFR^−/−^ mice than in WT mice in epididymal fat tissue at 12 weeks after the HFD (Fig. 6D). Immunofluorescence demonstrated that both EF M1 ATMs (TNF-α and IL-1β positivity) and M2 ATMs (CD206 and Arginase 1 positivity) were significantly lower in MΦ EGFR^−/−^ mice than WT mice (Fig. 6E, F).

**Fig. 7:**
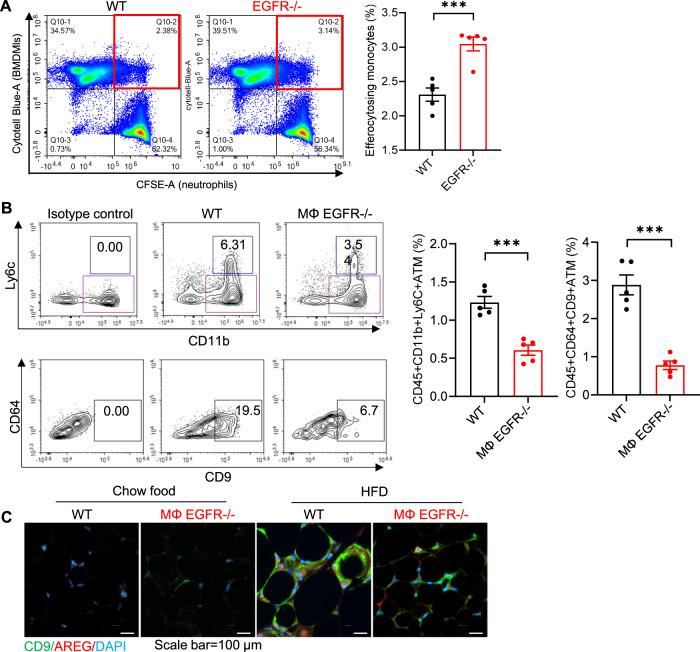
Myeloid EGFR^−/−^ mice had decreased subpopulations of ATMs in diet-induced obesity. **A** An efferocytosis assay was performed by ex vivo co-culturing BMDMs (labeled with Cytotell Blue) isolated from WT and myeloid EGFR^−/−^ mice with neutrophils isolated from WT mice that were treated with Staurosporine to induce apoptosis and labeled with CFSE. The first quadrant (in red) represents the efferocytosing monocyte population. Flow cytometry analysis showed that EGFR^−/−^ BMDMs had enhanced efferocytosis of apoptotic neutrophils, as indicated by a higher ratio of phagocytic to non-phagocytic cells. *N* = 4 and 5. **B** Flow cytometric analysis of epididymal fat ATMs (gating on CD45 + , Ly6G-, CD3-, CD4-, CD8-, SiglecF-) determined that both CD11b + , Ly6C + (Ly6C + ) ATMs and CD11b + , Ly6C-, CD64 + , F4/80 + , CD9 + (Ly6C-CD9 + ) ATMs were lower in MΦ EGFR^−/−^ mice than WT mice fed the HFD for 6 weeks. *N* = 5. **C** Double immunofluorescent staining determined that both CD9 and AREG expression was evident and colocalized in ATMs in CLSs in WT mice with HFD while their expression was lower in MΦ EGFR^−/−^ mice on HFD and minimal in both WT and MΦ EGFR^−/−^ mice on chow food. Scale bar = 100 μm. Data are means ± SEM, ***P < 0.001, analyzed using 2 tailed Student’s *t* test.

The ATM pool is made of macrophage subpopulations with distinct functions. Hill et al reported that both Ly6c^+^ ATMs and Ly6c^-^CD9^+^ ATMs are monocyte-derived, recruited and increased in obesity^22^. Ly6c^+^ ATMs reside outside of crown-like structures and have adipogenesis functions while Ly6c^-^CD9^+^ ATMs reside within crown-like structures and exhibit a proinflammatory and metabolically activated adipose tissue macrophage phenotype to clear apoptotic adipocytes^22–27^. EGFR^−/−^ BMDMs had increased phagocytic ability (Fig. 7A). Using flow cytometry analysis, we determined that there were both markedly fewer Ly6c^+^ ATMs and Ly6c^-^CD9^+^ ATMs in EF from MΦ EGFR^−/−^ mice compared to WT mice at 6 weeks after initiation of the HFD (Fig. 7B). Immunofluorescent staining confirmed that CD9 ATMs were primarily localized to CLSs from HFD mice, and AREG localized to CD9 ATMs in CLSs in EF. MΦ EGFR^−/−^ mice had fewer CD9 ATMs and fewer AREG-expressing ATMs (Fig. 7C). Therefore, EGFR deletion results in reduce accumulation of ATMs but with enhanced phagocytic ability.

With polarization to an M1 or M2 phenotype, MΦ EGFR^−/−^ BMDMs exhibited reduced induction of proinflammatory cytokine genes including *Ccl2*, *Nos2*, *Tnf*, *Irf5*, and *Il23a* (Supplementary Fig. 15B) but greater induction of anti-inflammatory cytokine genes including *Arg1*, *Cd206*, and *Ym1* (Supplementary Fig. 15C). M1 polarization increased (>5-fold increased) and M2 polarization inhibited (>80% inhibition) *Areg* mRNA levels in BMDM. AREG deletion in BMDMs also led to reduced induction of proinflammatory cytokine genes and greater induction of anti-inflammatory cytokine genes (Supplementary Fig. 16).

### Myeloid EGFR^−/−^ mice had less monocyte recruitment and ATM proliferation

**Fig. 8:**
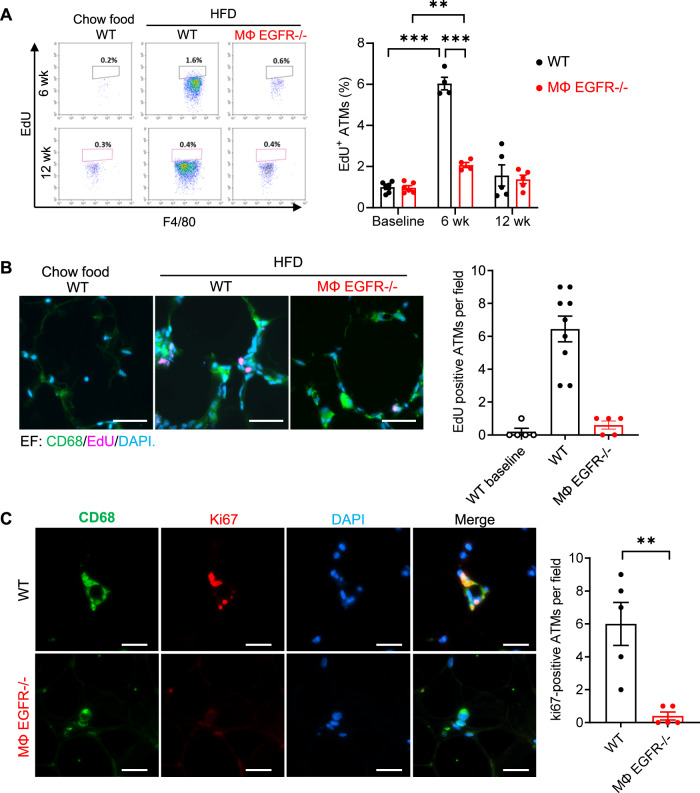
Mice with myeloid EGFR deletion had decreased ATM proliferation in diet-induced obesity. WT and MΦ EGFR^−/−^ mice were fed the HFD for 6 or 12 weeks. **A** The significantly increased proliferating rate of ATMs in EF (EdU positive cells) seen in WT mice at 6 weeks was markedly attenuated in MΦ EGFR^−/−^ mice. *N* = 4 and 5 and 6. **B**, **C** A decreased rate of proliferation in MΦ EGFR^−/−^ mice at 6 weeks was also indicated by decreased EdU and CD68-double positive cells (*n* = 5 and 9) **B** and Ki67 and CD68-double positive cells (*n* = 5) **C**. Scale bar = 100 μm. Data are means ± SEM, **P < 0.01, ***P < 0.001, analyzed using 2-way ANOVA followed by Bonferroni’s post hoc test for **A** and **B**, and 2 tailed Student’s t test for **C**.

Both increased monocyte recruitment and proliferation of resident ATMs can potentially contribute to ATM accumulation in response to the HFD. To investigate whether there were differences in ATM proliferation between MΦ EGFR^−/−^ mice and WT mice in obesity, Click-iT™ Plus EdU Alexa Fluor™ 647 was injected intraperitoneally 3 h before sacrifice to evaluate cells in the S phase of proliferation. Compared to normal chow-treated WT mice, the proliferation rate of ATMs in EF in 6-week HFD-treated mice was 6.04-fold higher in WT but only 2.08-fold higher in MΦ EGFR^−/−^; in contrast, there was no significantly increased ATM proliferation observed in either WT mice or MΦ EGFR^−/−^ mice after 12 weeks on the HFD (Fig. 8A), consistent with previous reports^1–4^. A decrease in proliferating ATMs (EdU and CD68-double positive) was confirmed by immunofluorescence in EF in MΦ EGFR^−/−^ mice compared to WT mice at 6 weeks of the HFD (Fig. 8B). Additionally, MΦ EGFR^−/−^ mice at 6 weeks of the HFD also had decreased percentages of ATMs that were positive for Ki67, which is expressed at all stages of cell proliferation from G1 through mitosis (Fig. 8C).

**Fig. 9:**
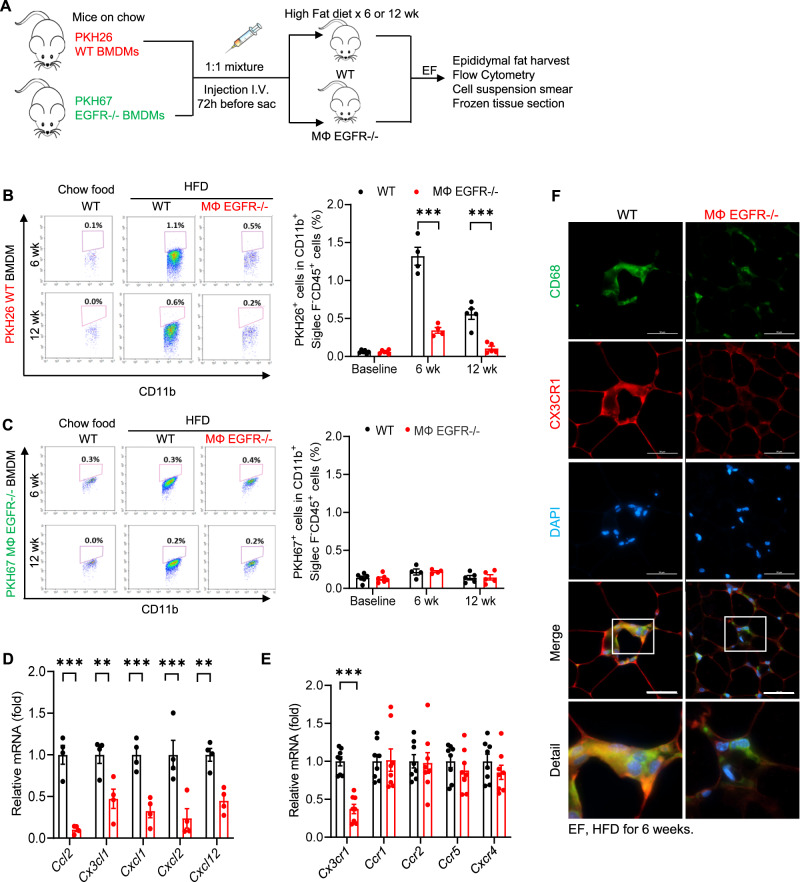
Both intrinsic EGFR in bone marrow derived monocyte (BMDMs) and adipose tissue from MΦ EGFR^−/−^ mice affected the recruitment of BMDMs in diet-induced obesity. WT and MΦ EGFR^−/−^ mice were fed the HFD for 6 or 12 weeks and EF was used for experiment. **A** Schematic of experimental protocol. **B** WT BMDMs had greater EF infiltration at 6 weeks than at 12 weeks in WT recipient mice. Infiltration into EF was markedly decreased in MΦ EGFR^−/−^ recipients at both time points. *N* = 4 and 5 and 6. **C** The infiltration of EGFR^−/−^ BMDMs in EF was not increased after high fat diet in either WT or MΦ EGFR^−/−^ recipients. *N* = 4 and 5 and 6. **D** MΦ EGFR^−/−^ mouse epididymal fat tissue had lower mRNA expression of chemoattractants, including *Ccl2, Cx3cl1*, *Cxcl1*, *Cxcl2*, and *Cxcl12*. *N* = 4. **E** ATMs isolated from MΦ EGFR^−/−^ mice had lower *Cx3cr1* mRNA expression. *N* = 8. **F** High CX3CR1 expression in ATMs seen in WT mice was decreased in MΦ EGFR^−/−^ mice 6 weeks after the HFD. Scale bar = 50 μm. Data are means ± SEM, *P < 0.05, **P < 0.01, ***P < 0.001, analyzed using 2-way ANOVA followed by Bonferroni’s post hoc test for all.

We employed the strategy depicted in Fig. 9A to investigate the role of myeloid EGFR in monocyte recruitment. We labeled WT BMDMs with the monocyte tracking dye PKH26 (red) and EGFR^−/−^ BMDMs with PKH67 (green) and injected a mixture containing equal amounts of labeled WT and EGFR^−/−^ BMDMs into WT or MΦ EGFR^−/−^ recipients fed the HFD for 6 weeks. We determined that the percentage of PKH26-positive cells (WT BMDM origin) in EF was significantly higher in 6-week and 12-week HFD-treated WT recipients compared to corresponding MΦ EGFR^−/−^ recipients at both time points (Fig. 9B). In contrast, the percentage of PKH67-positive cells (EGFR^−/−^ BMDM origin) in EF was very low and was indistinguishable between recipient groups at either time point (Fig. 9C). More PKH26-positive WT BMDMs were also observed in cell suspension smears of EF from WT recipients (Supplementary Fig. 14). In vitro migration assays confirmed that MΦ EGFR^−/−^ BMDMs had decreased CX3CL1-induced migration. Migration of WT BMDMs was also markedly attenuated by pharmacologic EGFR inhibition (Supplementary Fig. 15A). CX3CL1-induced BMDM migration was also inhibited by inhibition of PI3K and AKT activity, downstream signaling components of EGFR activation (Supplementary Fig. 15A).

After six weeks on the HFD, MΦ EGFR^−/−^ mouse EF had significantly lower mRNA expression of a panel of chemoattractants, including *Cx3cl1*, *Ccl2*, *Cxcl1*, *Cxcl2*, and *Cxcl12* (Fig. 9D). In isolated EF ATMs, *Cx3cr1* mRNA expression was markedly lower in MΦ EGFR^−/−^ mice than in WT mice after 6 weeks on the HFD (Fig. 9E). Double immunofluorescent staining showed that CX3CR1 was highly expressed in ATMs in WT mice but was decreased in MΦ EGFR^−/−^ mice after 6 weeks on the HFD (Fig. 9F). These results indicated that both lower expression of monocyte chemotactic cytokines in adipose tissue and reduced receptors for chemotactic cytokines on ATMs may contribute to decreased monocyte recruitment in MΦ EGFR^−/−^ mice in HFD-induced obesity.

To investigate whether signaling from adipocytes regulated the observed properties of myeloid cells, we cocultured adipocytes with a mouse macrophage-like cell line, RAW264.7. Conditional media (CM) from apoptotic adipocytes stimulated *Areg* and *Adam17* expression levels in RAW264.7 cells (Supplementary Fig. 17A, B). In addition, CM from apoptotic cells stimulated proinflammatory cytokine expression and inhibited anti-inflammatory cytokine expression (Supplementary Fig. 17C, D). In addition, coculture with apoptotic adipocytes increased migratory ability of the RAW264.7 cells (Supplementary Fig. 17E).

## Discussion

In the current studies, we found: (1) both male and female *Waved* 2 mice with global intrinsic EGFR tyrosine kinase deficiency exhibited less weight gain and less insulin resistance in HFD-induced obesity; (2) selective deletion of EGFR in primary insulin sensitive cell types (hepatocytes, adipocytes, or skeletal muscle) had no effect on insulin resistance induced by the HFD; (3) the HFD led to increased expression of EGFR and its ligand, amphiregulin, in ATMs in CLSs; (4) selective EGFR deletion in myeloid cells attenuated insulin resistance in HFD-induced obesity; (5) hyperinsulinemic-euglycemic clamps determined that mice with selective myeloid EGFR deletion developed less insulin resistance specifically in white adipose tissue in response to a high fat diet; (6) mice with selective myeloid EGFR deletion had fewer adipose tissue macrophages after the high fat diet as a result of both decreased monocyte recruitment and decreased proliferation of resident macrophages; and (7) EGFR^−/−^ BMDMs have decreased migratory ability but increased phagocytic ability. Indirect calorimetry measured after 4 weeks suggest that differences in weight gain seen at this time were the result of increased activity by the MΦ EGFR^−/−^ mice. However, after 12 weeks on the high fat diet, the MΦ EGFR^−/−^ mice had relatively more brown fat than wild type mice, suggesting an additional etiology for the differences in weight gain.

Recent studies in experimental animals have demonstrated that ATM accumulation in white adipose tissue in the early stage of obesity is largely the result of in situ proliferation of resident ATMs, and the proliferating ATMs mainly reside in the CLSs. In obesity, ATM proliferation is only observed in adipose tissue and not in liver or spleen, indicating that the adipose tissue provides a unique environment facilitating macrophage proliferation in obesity^4^. Proliferating ATMs in CLS have also been confirmed in obese patients^2^. In WT mice, markedly increased resident ATM proliferation was only observed at 6 weeks after high fat feeding, in accordance with previous reports that resident ATM proliferation occurs at early stages of obesity^1–4^. However, ATM proliferation was decreased in MΦ EGFR^−/−^ mice. CCL2/MCP-1 not only acts as a potent chemoattractant but can also stimulate ATM proliferation^4^. Expression of CCL2 was markedly decreased in adipose tissue in MΦ EGFR^−/−^ mice, suggesting that decreased adipose tissue CCL2 might also contribute to the decreased ATM proliferation seen in MΦ EGFR^−/−^ mice.

As obesity proceeds, monocyte recruitment becomes more important^1–4^. In the current study, we confirmed that WT BMDM recruitment was markedly increased in WT recipients at 6 and 12 weeks after the high fat feeding, and this recruitment was markedly attenuated in recipients with selective myeloid EGFR deletion. In adipose tissue, the expression of a panel of monocyte chemoattractants, including Ccl2, Ccl3, Cx3cl1, Cxcl2, Cxcl2, Cxcl12, were all markedly lower in MΦ EGFR^−/−^ mice than WT mice at 6 weeks after the high fat feeding. Gene expression of a number of adipokine gene expression was also decreased, including not only leptin and resistin mRNA but also somewhat surprisingly, adiponectin mRNA. We attribute this decrease to the relative lack of adipose tissue in the MΦ EGFR^−/−^ mice.

We also observed that recruitment of EGFR deficient BMDMs was not increased in either WT recipients or recipients with myeloid EGFR deletion at either 6 weeks or 12 weeks after high fat feeding, suggesting an intrinsic recruitment impairment. In isolated ATMs from epididymal fat, we found that the expression of CX3CR1, the receptor for the chemoattractant CX3CL1, was markedly lower in ATMs isolated from MΦ EGFR^−/−^ mice than WT mice. Of note, CX3CL1/fractalkine is a well-described monocyte chemoattractant^28^. Therefore, both decreased fat tissue chemoattractants and decreased ATM receptor expression could contribute to decreased monocyte recruitment in MΦ EGFR^−/−^ mice.

The EGFR ligand amphiregulin has emerged as a potent mediator of inflammation. Amphiregulin has been reported to increase myeloid cell recruitment, polarize them to a proinflammatory M1 phenotype, and protect against apoptosis^29^. Mice lacking hematopoietic amphiregulin or mice with selective EGFR deletion in myeloid cells had diminished macrophage infiltration in glomerulonephritis^29^. In addition, amphiregulin is sufficient to induce macrophage recruitment in mammary glands^30^. In the present study, we found that amphiregulin mRNA levels increased more than 10 fold in epididymal fat at 6 weeks after the HFD while expression of other EGFR ligands was not altered, and amphiregulin expression was markedly decreased in ATMs from MΦ EGFR^−/−^ mice. ADAM17 mediates ectodomain shedding of amphiregulin in macrophages^31^, and in preliminary results, we also determined that ADAM17 mRNA was significantly decreased in ATMs from MΦ EGFR^−/−^ mice.

The increased amphiregulin expression seen in adipose tissue from wild type mice was primarily confined to ATMs, and amphiregulin and CD9 were colocalized in ATMs in CLSs. The group 2 innate lymphoid cells are resident cells in adipose tissue that also express amphiregulin^32^. In contrast to ATMS, ILC2s are decreased in HFD-induced obesity, suggesting a possible adaptive or protective role due to decreasing paracrine activation of EGFR in ATMs^24, 33, 34^.

Therefore, in WT mice, increased amphiregulin could contribute to ATM proliferation and polarization through activation of EGFR as well as promoting adipocyte dysfunction^35–45^. In this regard, global AREG deletion protected against insulin resistance in response to the HFD, and AREG^−/−^ BMDMs had impaired migration and reduced induction of proinflammatory genes with M1 polarization and greater induction of anti-inflammatory genes with M2 polarization.

We queried available scRNAseq data sets and found that *AREG* transcripts were expressed in macrophages in human adipose tissues, and macrophage *AREG* mRNA levels were higher in diabetic adipose tissue than in non-diabetic adipose tissue (Supplementary Fig. 18)^27^. Similarly, *Areg* transcripts were expressed in macrophages in mouse adipose tissues, and *Areg* mRNA levels were restricted to two specific subpopulations of ATMs and were higher in fat mice than in lean mice (Supplementary Fig. 19)^46^. In both data sets, EGFR mRNA was expressed in macrophages in adipose tissues, but no apparent increases were observed in diabetic adipose tissue compared to non-diabetic adipose tissue, which may be explained by the fact that EGFR activity depends on ligand binding and subsequent phosphorylation rather than expression per se.

EGFR activation is a known promoter of tumorigenesis in a variety of cancers and EGFR tyrosine kinase inhibitors and blocking antibodies are mainstays of treatment. Interestingly, inflammation also contributes to tumorigenesis^47^, and obesity is a strong risk factor for multiple cancer types. Obesity-related cancers are more aggressive and have shortened latency. In addition, weight gain itself is a strong cancer risk factor. Obesity has been attributed to 13 cancers, including cancers with EGFR inhibitors as a therapeutic strategy^48^. Therefore, in addition to providing direct evidence for a role in diet-induced obesity, our findings suggest that treatments that inhibit EGFR activity may not only directly inhibit tumor growth but could have an additional effect to improve metabolic homeostasis.

In summary, these results demonstrate that EGFR expression and activation in myeloid-derived cells plays an important detrimental role in the development of insulin resistance in obesity. Future studies will be required to determine if selective inhibition of amphiregulin expression could provide a therapeutic option to limit the detrimental consequences of obesity.

## Methods

### Animals

EGFR floxed *(*EGFR^f/f^*)* mice were generated by flanking exons 3 of the EGFR gene with two LoxP sites, as described previously^49^. CD11b-Cre mice with transgene integration in the Y chromosome were generated in Dr. Vacher’s laboratory^50^. Alb1-Cre mice (Stock No: 016832), Adipoq-Cre mice (Stock No:010803), and ACTA1-Cre mice (Stock No: 006149) were purchased from the Jackson Laboratory. We generated mice with selective EGFR deletion in myeloid cells (CD11b-Cre; EGFR^f/f^, MΦ EGFR^−/−^), hepatocytes (Alb1-Cre; EGFR^f/f^, Hepatocyte EGFR^−/−^), adipocytes (Adipoq-Cre; EGFR^f/f^, Adipocyte EGFR^−/−^), and skeletal muscle (ACTA1-Cre; EGFR^f/f^, smEGFR^−/−^), and EGFR^f/f^ mice were used as WT controls. All these mice were on a C57BL/6 background. *Waved 2* mice harboring a point mutation in the EGFR gene, with resultant loss of most of its tyrosine kinase activity (Stock No: 025148)^51^, were also purchased from The Jackson Laboratory, and both male and female 8–12 weeks old *waved 2* (*wa2/wa2*) and littermate *wa2/+* (control) were used. The primers used in the current studies are listed in Supplementary Table 1. C57/B6 AREG^−/−^ mice were originally generated by Dr. Lee^52^.

### Antibodies and reagents

The information of all antibodies used is listed in Supplementary Table 2. The HFD was from Bio-Serv (36% fat accounting for 60% of calories, #F3282, Frenchtown, NJ, USA).

### Isolation of bone marrow neutrophil, BMDMs and ex vivo polarization, and isolation of fat tissue myeloid cells

The mice were anesthetized with isoflurane and sacrificed by cervical dislocation. Femurs, tibias, and humeri were dissected, and the shafts were flushed using a syringe and a 26-gauge needle with RPMI1640 supplemented with 100 U/ml penicillin, 100 μg/ml streptomycin, 10 U/ml heparin, and 0.2% fetal bone serum (FBS). The cell suspension was passed through a 40-μm strainer, centrifuged, and the pellets were resuspended in 3 ml red blood cell lysis buffer and incubated for 3 min at RT. After centrifugation, the pellets were resuspended with 10 ml Dulbecco’s PBS containing 0.5% FBS, followed by monocyte isolation using a monocyte isolation kit for mice (Miltenyi Biotec, 130-100-629)^53^. An M0 phenotype was achieved by culturing BMDMs in DMEM for 48 h. An M1 or M2 polarization was achieved by culturing with LPS and IFN-γ (1 μg/ml for each) for 24 h or IL-4 and IL-13 (10 ng/ml for each) for 48 h, respectively. Neutrophils were isolated by anti-Ly-6G microbeads (130-120-337, Miltenyi) from bone marrows of WT mice. Adipose tissue myeloid cells were enriched using mouse F4/80 Microbeads and MACS columns (Milteni Biotec Auburn, CA) following the manufacturer’s protocol.

### In vitro migration assay

Migration assays were performed as previously described^54^. Freshly isolated BMDMs (100,000 cells) were seeded in the top chamber of a 24-well PET membrane (8 mm pore size). Cells translocated to the lower chamber in response to exposure in the lower chamber of vehicle or 100 ng/ml CX3CL1 (472-FF; R&D) in the presence or absence of either an AKT inhibitor (MK-2206; 10 μM, Cayman), PI3K inhibitor (BEZ235; 10 μM, Cayman), EGFR inhibitor (erlotinib, 10 μM, LC laboratories) or NF-κB inhibitor (JSH23, 30 μM, Sigma-Aldrich) for 3 h. Cells in the upper chamber were removed with a cotton swab, and the filters were fixed with 70% ethanol and stained with 2% crystal violet. Filters were photographed on a Leica DMi1 microscope and total cell number was counted.

### Crosstalk between adipocytes and macrophages

3T3-L1 cells (ATCC® CL-173™) were differentiated into adipocyte-like cells with IBMX-DEX-INS medium for 10 days and induced to apoptosis by a staurosporine apoptosis inducer (1:1000). For a migration assay, 1 × 10^5^ mouse macrophage-like RAW264.7 cells (ATCC® TIB-71™) were layered on the upper chamber of a 24-well PET membrane in serum-free medium, then transferred to a new well containing either serum-free medium or apoptotic adipocytes with serum-free medium for 16 h. For polarization, RAW264.7 cells were cultured in serum-free medium or medium from 3 h of apoptotic adipocytes for 16 h.

### Hyperinsulinemic-euglycemic clamp

Mice were anesthetized under 2% (v/v) isoflurane and the left common carotid artery and the right jugular vein were catheterized for sampling and infusions, respectively, as previously described^15^. On the day of the experiment, food was removed at 8:00 A.M. After 3.5 h fasting, a primed bolus (2 min, 0.5 μCi/min) followed by continuous infusion (0.05 μCi/min) of [3-^3^H] glucose was administered to measure whole-body glucose turnover. After 5 h fasting, mice received a continuous insulin infusion (4 mU/kg/min), and blood glucose was maintained at basal levels by a variable infusion of a 50% (w/v) glucose solution. Arterial blood samples were collected during steady-state conditions and at 80, 90, 100, 110, and 120 min for determination of Rd and Ra, as described above. At 120 min, a 13 μCi bolus of [14 C]−2-deoxy-D-glucose (2-DG) was injected into the jugular vein, and arterial blood was sampled at 122, 135, 145, 155 min. Mice were administered pentobarbital anesthesia and tissues were extracted and frozen for subsequent gene expression and glucose uptake determinations.

### Flow cytometry and efferocytosis assay

Epididymal fat tissue cell suspension was prepared using DNAse1 (56 U/ml, Bio-Rad#7326828) and collagenase D (4 mg/ml, Roche#11088882001), and then suspended in 100 μl of PBS containing 1% BSA and incubated with 0.5 μl Fc block (BD Pharmingen, purified Rat anti-Mouse CD16/CD32. Anti-CD45, anti-CD11b, and anti-F4/80 were used to identify ATMs (Supplementary Table 2). All these reagents were from BioLegend. Appropriate isotype controls were included for each sample. A total of 100,000 cells were acquired by scanning using NovoCyte Quanteon Flow Cytometer Systems. Cell debris and dead cells were excluded from the analysis based on scatter signals and use of Zombie Violet™ Fixable Viability Kit (Cat#423114, Biolegend). For evaluation of monocyte infiltration into adipose tissue, 100 μl of solution containing 10^6^ PKH26-labeled WT BMDMs (red, PKH26GL, Sigma-Aldrich) and 10^6^ PKH67-labeled EGFR^−/−^ BMDMs (from MΦ EGFR^−/−^ mice) (green, PKH67GL, Sigma-Aldrich) were injected retro-orbitally to either WT or MΦ EGFR^−/−^ mice on the HFD for 6 weeks, 72 h before sacrifice. Cell proliferation in S phase was evaluated using Click-iT™ Plus EdU Alexa Fluor™ 647 Flow Cytometry Assay Kit (Invitrogen™, C10634), which was administrated via intraperitoneal injection 3 h before sacrifice according to the manufacturer’s protocol.

The efferocytosis assay was performed using an efferocytosis assay kit (NO.601770, Cayman Chemical) following the manufacturer’s protocol. Isolated neutrophils from WT mice were stained with CFSE and then treated with Staurosporine (1:1000) for 2 h at 37 °C to induce apoptosis. Isolated BMDMs from WT and myeloid EGFR-/- mice were stained with CytoTell^TM^blue, plated onto 6-well plates (3 × 10^5^ cells/well), and incubated with labeled apoptotic neutrophils (1:4) at 37 °C for 16 h. For detection of efferocytosis, cells were gated on CFSE positivity and CytoTell^TM^ blue positivity before counting the number of events that were both CFSE and CytoTell^TM^ blue positive.

### Intraperitoneal glucose tolerance test (GTT) and insulin tolerance test (ITT)

For GTT, mice fasted overnight (16 h) were intraperitoneally injected with glucose at a dose of 2 mg/g body weight and blood glucose was measured 0, 15, 30, 90, 120, 150, and 180 min after glucose injection^8^. For ITT, insulin was given at a dose of 3 µg/kg body weight to mice fasting from 8:00 a.m. to 2:00 p.m., and blood glucose was monitored 0, 15, 30, 45, 60, 75, and 90 min after insulin administration^8^. Blood glucose was evaluated with a B-Glucose Analyzer (HemoCue, Lake Forest, CA).

### Quantitative PCR

Total RNAs from tissues and cells were isolated using Trizol® reagent (Invitrogen). SuperScript IV First-Strand Synthesis System kit (Invitrogen) was used to synthesize cDNA from equal amounts of total RNA from each sample. Quantitative RT-PCR was performed using TaqMan real-time PCR (7900HT, Applied Biosystems). The Master Mix and all gene probes were also purchased from Applied Biosystems. The probes used in the experiments are listed in Supplementary Table 3. Realtime PCR data were analyzed using the 2-ΔΔCT method to determine the fold difference in expression.

### Quantitative immunofluorescence staining

Animals were anesthetized with Nembutal (70 mg/kg, i.p.) and given heparin (1000 units/kg, i.p.) to minimize coagulation. After perfusion with cold PBS through a transcardial aortic cannula, tissues were removed for immunoblotting, flow cytometry, qPCR, immunofluorescent staining, isolation of ATMs, or histology with immersion in fixative containing 3.7% formaldehyde, 10 mM sodium m-periodate, 40 mM phosphate buffer, and 1% acetic acid. To determine insulin sensitivity in different organs with p-Akt immunoblotting and immunofluorescent staining, insulin at a dose of 1.5 u/kg was given intraperitoneally 5 min before sacrifice. The fixed tissue was dehydrated through a graded series of ethanols, embedded in paraffin, sectioned (5 μm), and mounted on glass slides. The deparaffinized sections underwent antigen retrieval with citrate buffer by microwave heat for 10 min, and then were blocked with 10% normal donkey serum for 1 h at room temperature. For double immunofluorescence staining, the sections were incubated in two rounds of staining: CD68 vs. EGFR, TNF-α, IL-1β, CD206, ARG1, or amphiregulin, overnight at 4 °C, followed by either anti-rabbit IgG-HRP or anti-mouse IgG-HRP incubation at room temperature for 1 h. Each round was followed by tyramide signal amplification with Alexa Flour 488 tyramide or Alexa Flour 555 tyramide (Tyramide SuperBoost Kit with Alexa Fluor Tyramides, Invitrogen) according to its manufacturer’s protocols. DAPI was used as a nuclear stain. Of note, 5 μm cryo-sections from fresh tissue embedded by Tissue-Tek OCT were used for CD68 vs. EdU and CD68 vs. Ki67 immunofluorescent staining in fat tissues. Sections were viewed and imaged with a Nikon TE300 fluorescence microscope and spot-cam digital camera (Diagnostic Instruments), followed by quantification using Image J software (NIH, Bethesda, MD) in more than 10 fields per slide and expressed as arbitrary units or percentage per field by two independent investigators.

### Immunoblotting analysis

Livers and epididymal fat tissue were homogenized using lysis buffer containing 10 mmol/l Tris–HCl (pH 7.4), 50 mmol/l NaCl, 2 mmol/l EGTA, 2 mmol/l EDTA, 0.5% Nonidet P-40, 0.1% SDS, 100 μmol/l Na3VO4, 100 mmol/l NaF, 0.5% sodium deoxycholate, 10 mmol/l sodium pyrophosphate, 1 mmol/l PMSF, 10 μg/ml aprotinin, and 10 μg/ml leupeptin and centrifuged at 15,000 × *g* for 20 min at 4 °C^55^. Adipose tissue protein was extracted with Minute™ Total Protein Extraction Kit (Invent biotechnologies). The BCA protein assay kit (Thermo Scientific) was used to measure the protein concentration. Immunoblotting was performed as previously described^55, 56^ and quantitated with Image J software.

### Hemoglobin A1c (HbA1c) evaluation

HbA1c levels were measured using DCA Vantage Analyzer (SIEMENZ).

### Indirect calorimetry

Mice were individually placed in home-cages in a 12 h light/dark cycle, temperature/humidity-controlled dedicated room located in the Vanderbilt MMPC (RRID: SCIR_021939). Energy expenditure measures were obtained by indirect calorimetry (Promethion, Sable Systems, Las Vegas, NV)^17^. The calorimetry system consists of home cages with bedding equipped with water bottles and food hoppers connected to load cells for food and water intake monitoring. All animals had ad libitum access to standard chow or high fat diet and water. The air within the cages is sampled through microperforated stainless steel sampling tubes that ensure uniform cage air sampling. Promethion utilizes a pull-mode, negative pressure system with an excurrent flow rate set at 2000 mL/min. Water vapor is continuously measured and its dilution effect on O2 and CO2 are mathematically compensated for in the analysis stream. O2 consumption and CO2 production are measured for each mouse every 5 min for 30 s. Incurrent air reference values are determined every 4 cages. Respiratory quotient (RQ) is calculated as the ratio of CO2 production over O2 consumption. Energy expenditure is calculated using the Weir equation: EE (kcal/h) = 60*(0.003941*VO2(ml/min) +0.001106*VCO2(ml/min). Ambulatory activity was determined every second with XYZ beams. Data acquisition and processing were coordinated by MetaScreen and MacroInterpreter (Sable Systems). Body composition was determined by NMR (Bruker Minispec).

### Statistical analysis

Statistical analyses were performed with GraphPad Prism 9 (GraphpadSoftware® Inc., La Jolla, CA, US). Data are presented as the mean ± S.E.M. Data were analyzed using 2 tailed Student’s *t* test, two-way ANOVA followed by Tukey’s or Bonferroni’s post hoc tests. A *P* value less than 0.05 was considered significant. For each set of data, at least 4 different animals were examined for each condition. Collection, analysis, and interpretation of data were conducted by at least 2 independent investigators, who were blinded to the study.

### Study approval

All animal experiments were performed in accordance with the guidelines and approval of the Institutional Animal Care and Use Committee of Vanderbilt University.

### Reporting summary

Further information on research design is available in the Nature Research Reporting Summary linked to this article.

## Supplementary information

Supplementary information

Reporting Summary

## Source data

Source Data
