# Supplementary material for: EGFR-mediated activation of adipose tissue macrophages promotes obesity and insulin resistance
Source: Nat Commun. 2022 Aug 10;13:4684. doi: 10.1038/s41467-022-32348-3 (PMC9365849; doi:10.1038/s41467-022-32348-3)
Supplement: Supplementary file 1 — Supplementary information [file 41467_2022_32348_MOESM1_ESM.pdf]

Supplemental *Appendix,* Table 1. List of primers for genotyping.

Alleles/Transgenes	Primer
Waved 2	wave2-F: 5′-ATAACCTGACACTTGTCAGAGTAC-3′<br>wave2-R: 5′-TTTGCAATCTGCACACACCAGTTG-3′
EGFR-flox	EGFR-S: 5′-CTTTGGAGAACCTGCAGATC-3′<br>EGFR-AS: 5′-CTGCTACTGGCTCAAGTTTC-3′
CD11b-Cre	CD11b-CreF: 5’-AATGCTTCTGTCCGTTTGC-3’<br>CD11b-CreR: 5’- CGGCAACACCATTTTTTCTG-3’
ACTA1-Cre	oIMR1084: 5’-GCGGTCTGGCAGTAAAAACTATC-3’<br>oIMR1084: 5’- GTGAAACAGCATTGCTGTCACTT-3’<br>oIMR 7338: 5’- CTAGGCCACAGAATTGAAAGATCT-3’<br>oIMR 7339: 5’- GTAGGTGGAAATTCTAGCATCATCC-3’
Adipoq-Cre	Adipoq-CreF: 5’-ACGGACAGAAGCATTTTCCA-3’<br>Adipoq-CreR: 5’-GGATGTGCCATGTGAGTCTG-3’
Alb1-Cre	Albumin-CreF: 5′-ACCTGAAGATGTTCGCGATTATCT-3’<br>Albumin-CreR: 5′-ACCGTCAGTACGTGAGATATCTT-3’
AREG-/-	AR F1M: 5’-CTTTCCAGCTTTCTCCACCTCAAG -3’<br>AR R1M: 5’-ACAGTAACCTCTGTTGCATGCCAC-3’<br>PGK2: 5’-CTGCACGAGACTAGTGAGACGTGC-3’

Supplemental Appendix, **Table 2.** List of antibody, source, application and dilution

Antibody	Catalog Number	Dilution
Goat anti-EGFR	Sigma, E1281	IF 1:50
Rabbit anti-Amphiregulin	Abcam, ab234750	WB 1:1000; IF 1:100
Rabbit anti-Perilipin-1	Cell signal technology, 9349	IF 1:100
Rabbit anti-IL-1β	Abcam, ab9722	IF 1:100
Rabbit anti-Ki67	Abcam, ab16667	IF 1:300
Rat anti-F4/80	Bio-Rad, MCA497R	IF 1:50
Rabbit anti-CD68	Abcam, ab125212	IF 1:100
Rabbit anti-CD206	Proteintech, 18704-1-AP	IF 1:100
Rabbit anti-Arginase 1	Novus biologicals, NBP1-32731	IF 1:100
Rabbit anti-p-AKT	Cell signal technology, 4046	WB 1:1000; IF 1:100
Rabbit anti-AKT	Cell signal technology, 4685	WB 1:1000
Mouse anti-α-Tubulin	Cell signal technology, 3873	WB 1:2000
Mouse anti-TNF-a	Santa Cruz, SC-133192	IF 1:50
Rabbit anti-CX3CR1	Abcam, ab8021	IF 1:100
Anti-mouse CD45 (30-F11)	Biolegend 103149	FC 0.2 mg/ml
Anti-mouse/human CD11b (M1/70)	Biolegend 101226	FC 0.2 mg/ml
Anti-mouse F4/80 (BM8)	Biolegend 123130	FC 0.2 mg/ml
Purified anti-mouse CD16/32 (93)	Biolegend 101302	FC 0.2 mg/ml
Anti-mouse CD170 (1RNM44N)	eBioscience, 56170280	FC 0.2 mg/ml
Anti-mouse Ly-6G (1A8)	Biolegend 127614	FC 0.2 mg/ml
Anti-mouse CD9 (MZ3)	Biolegend 124808	FC 0.2mg/ml
Anti-mouse CD64 (X54-5/7.1)	Biolegend 139308	FC 0.2mg/ml
Anti-mouse CD170 (S17007L)	Biolegend 155506	FC 0.2mg/ml
Anti-mouse CD8a (53-6.7)	Biolegend 100708	FC 0.2mg/ml
Anti-mouse CD19 (6D5)	Biolegend 115508	FC 0.2mg/ml
Anti-mouse CD3e (145-2C11)	Biolegend 100308	FC 0.2mg/ml
Anti-mouse Ly6c (HK 1.4)	Biolegend, 128024	FC 0.2mg/ml
Rabbit anti-CD9	ThermoFisher, MA531980	IF 1:50

***Supplemental Appendix,* Table 3.** List of gene probes (Applied Biosystems)

Mouse primer	Catalog Number
Emr1	Mm00802529
Cd68	Mm03047343
Tnf	Mm99999068
Il1b	Mm00434228
Il6	Mm00446190
Ccl2	Mm00441242
Col1a1	Mm00801666
Col3a1	Mm01254476
Col4a1	Mm01210125
Acta2	Mm01546133
Tgfb1	Mm00441726
Ctgf	Mm01192933
Nos2	Mm00440502
Cd206	Mm01329362
Arg1	Mm00475991
Il4ra	Mm01275139
Cd209	Mm00460067
Ccl2	Mm00441242
Ccl3	Mm00441258
Ccr1	Mm01216147
Ccr2	Mm01216173
Ccr5	Mm04207879
Cx3cl1	Mm00436454
Cxcl12	Mm00445553
Cxcl1	Mm04207460
Cxcl2	Mm00436450
Cx3cr1	Mm00438354
Cxcr4	Mm01292123
Egfr	Mm00433023
Erbb2	Mm00658541
Erbb3	Mm01159999
Erbb4	Mm01256793
Egf	Mm01316968
Tgf a	Mm00446232
Hb-egf	Mm00439307
Areg	Mm00437583
Btc	Mm00432137
Ereg	Mm00514794
Epgn	Mm00504344
Lep	Mm00434759
Adipoq	Mm00456425
Hif1a	Mm00468869
Pgk1	Mm00435617
Ldha	Mm01612132

Glut1	Mm00441477
Hk1	Mm00439344
Pkm	Mm00834102
Pfdb3	Mm00504650
Ucp1	Mm01244861
Acta2	Mm01546133
Irf5	Mm00496477
Il23a	Mm00518984
Ym1	Mm00657889
Fizz1	Mm00445109

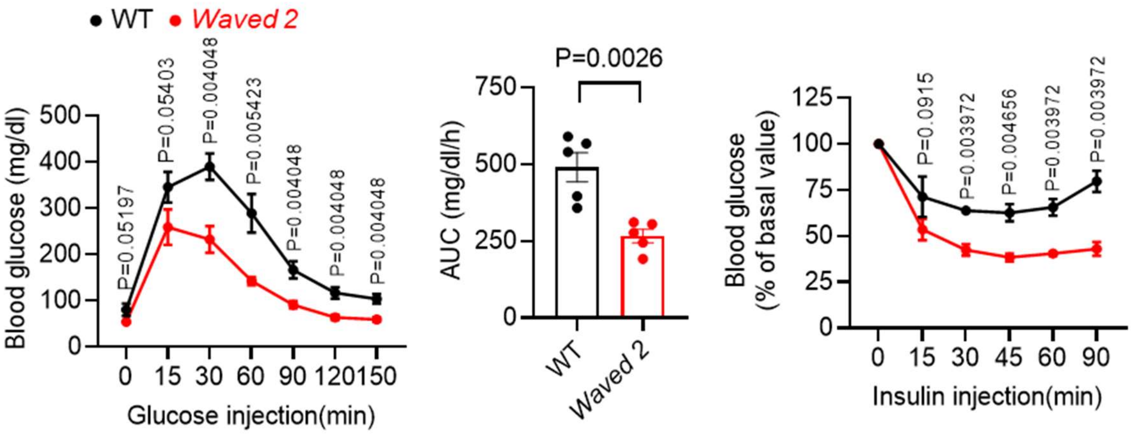

Supplementary Figure 1. Female *Waved* 2 mice developed less insulin resistance in diet-induced obesity. Female *Waved* 2 mice with intrinsic EGFR tyrosine kinase deficiency and WT mice were fed the HFD for 12 weeks. Female *Waved 2* mice had improved glucose tolerance test (GTT, n=5) and insulin tolerance test (ITT, n=3)**.**

Data are means ± SEM, analyzed using 2 tailed Student's t test and 2-way ANOVA followed by Tukey's post hoc test for GTT and 2-way ANOVA followed by Tukey's post hoc test for ITT.

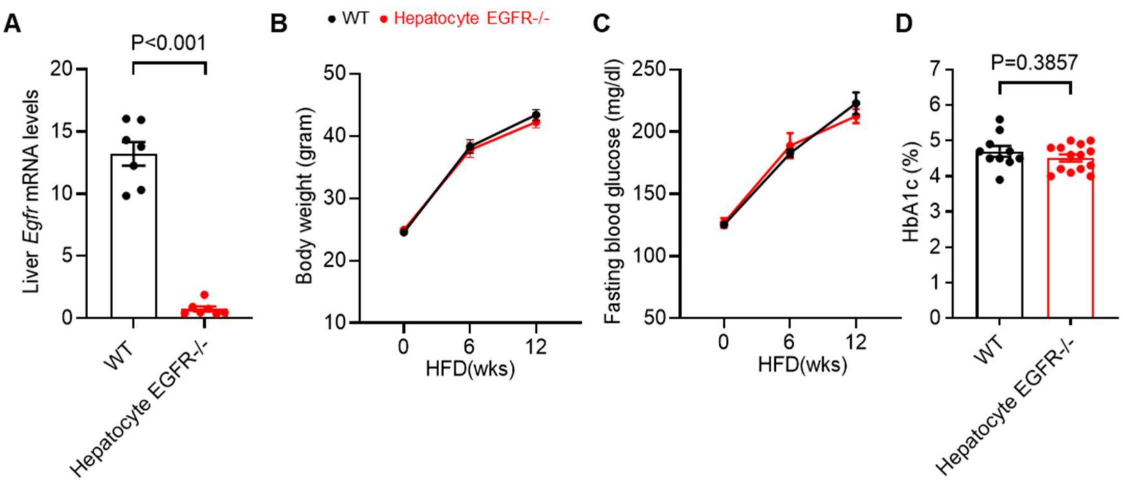

Supplementary Figure 2. Selective EGFR deletion in hepatocytes had on effect on insulin resistance in diet-induced obesity. Male hepatocyte EGFR-/- (Alb1-Cre; EGFR$^{f/f}$) mice and WT (EGFR$^{f/f}$) mice were fed with the HFD for 12 weeks. (**A**) Hepatocyte EGFR-/- mice had markedly lower liver *Egfr* mRNA levels compared to WT mice. N=7. (**B** and **C**) Hepatocyte EGFR-/- mice and WT mice had similar body weight gain (**B**) and fasting blood glucose (**C**) after the HFD treatment. N=10 and 14. (**D**) Hepatocyte EGFR-/- mice and WT mice had similar HbA1c at 12 weeks after the HFD. N= 10 and 14.

Data are means ± SEM, analyzed using 2 tailed Student's t test for **A** and **D**; 2-way ANOVA followed by Tukey's post hoc test for **B** and **C**.

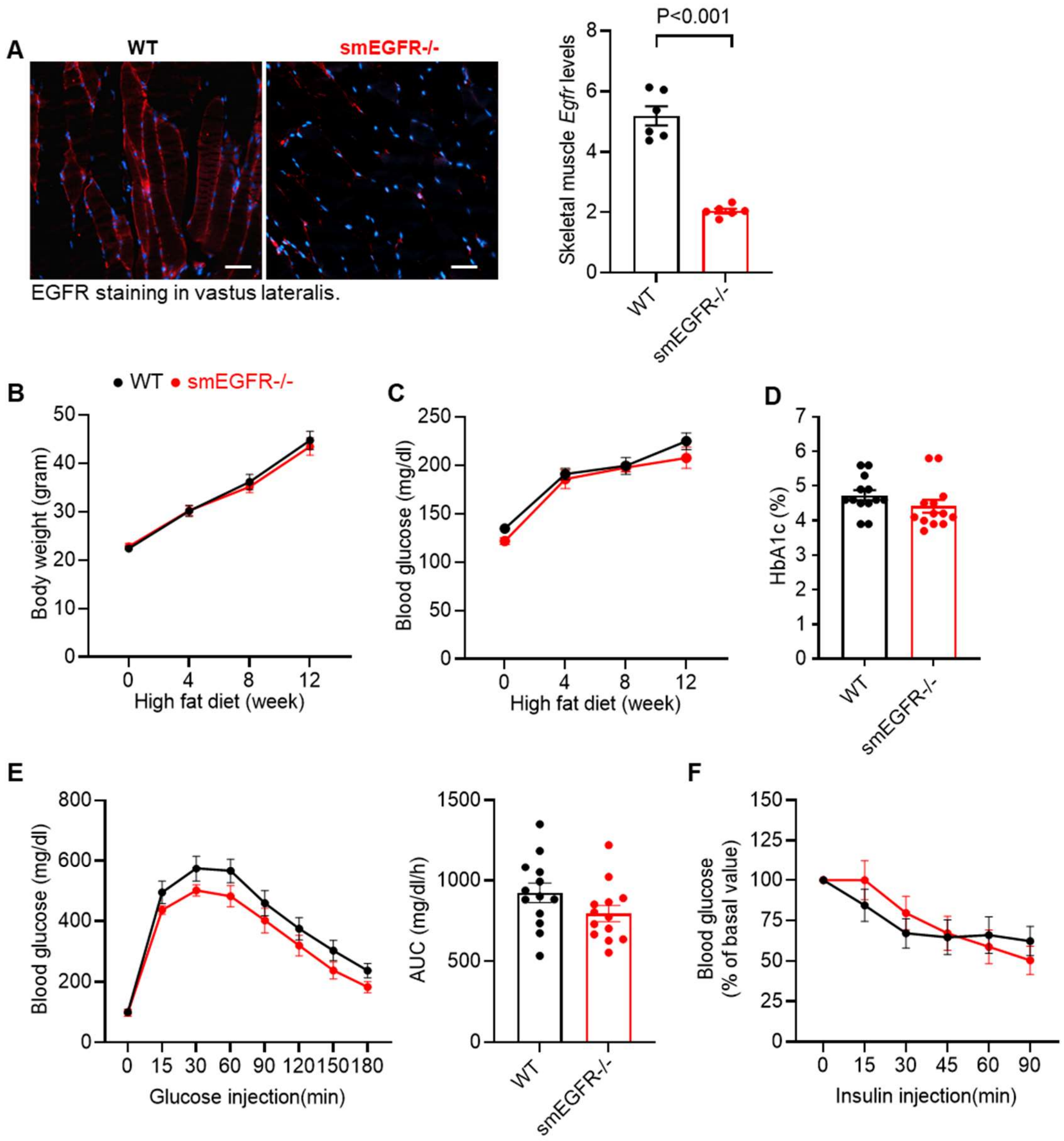

Supplementary Figure 3. Selective deletion of EGFR in skeletal muscle had on effect on insulin resistance in diet induced obesity. Male smEGFR-/- (ACTA1-Cre; $EGFR^{f/f}$) mice and WT ($EGFR^{f/f}$) mice were fed with the HFD for 12 weeks. (**A**) Both EGFR mRNA and protein levels in vastus lateralis were decreased in smEGFR-/- mice compared to WT mice. N=6. Scale bar=100μm. (**B-D**) smEGFR-/- mice and WT mice had similar body weight gain (**B**), fasting blood glucose (**C**), and HbA1c (**D**). N=13. (**E** and **F**) smEGFR-/- mice and WT mice had similar glucose tolerance test (GTT, n=13) (**E**) and insulin tolerance test (ITT, n=7) (**F**).

Data are means ± SEM, analyzed using 2 tailed Student's t test for **A and D**, 2-way ANOVA followed by Tukey's post hoc test for **B, C**, and **F;** and 2 tailed Student's t test and 2-way ANOVA followed by Tukey's post hoc test for **E**.

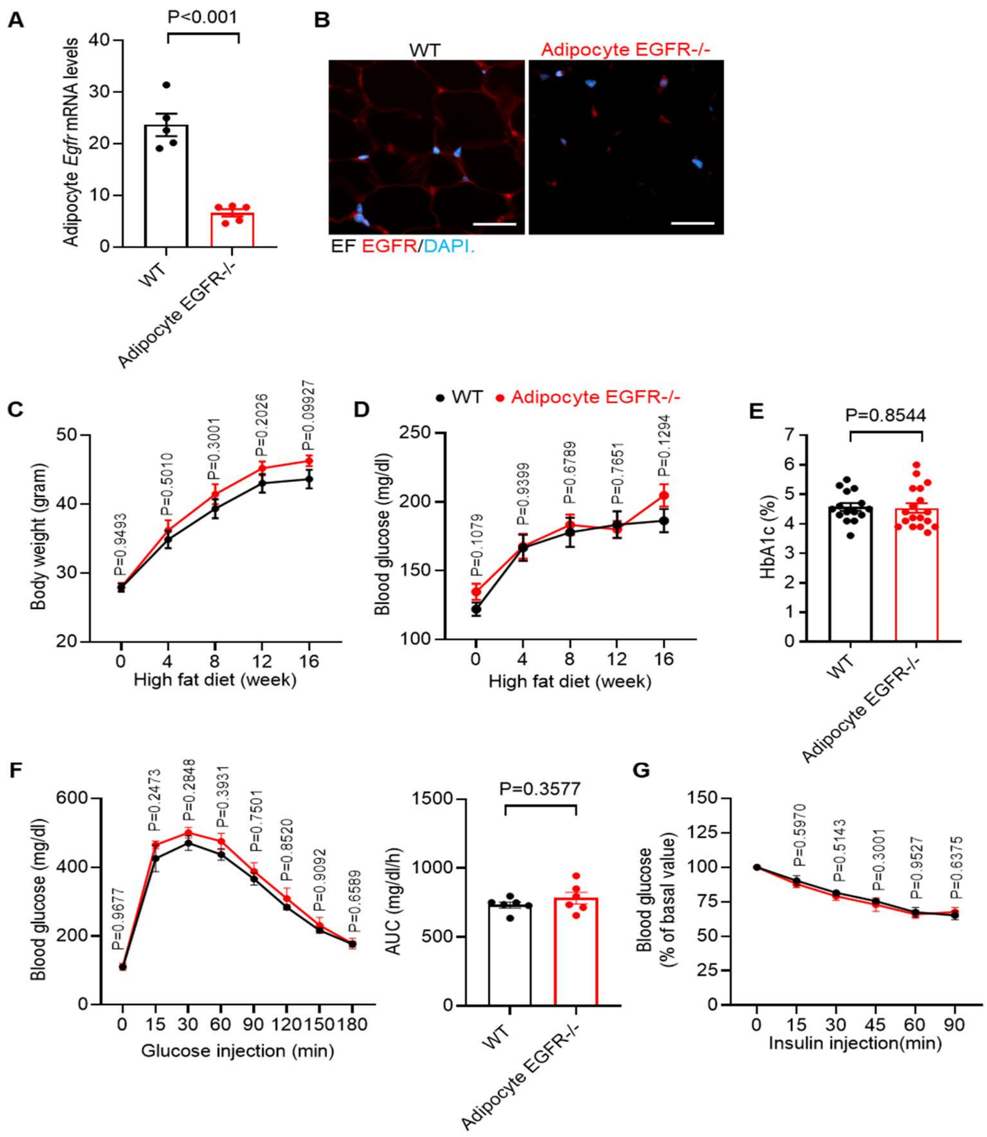

Supplementary Figure 4. Selective deletion of EGFR in adipocytes in male mice had on effect on insulin resistance in diet induced obesity. Male Adipocyte EGFR-/- (Adipoq-Cre; EGFR$^{f/f}$) mice and WT (EGFR$^{f/f}$) mice were fed with the HFD for 16 weeks. (**A and B**) EF EGFR mRNA (**A**) and protein (**B**) expression was markedly decreased in adipocyte EGFR-/- mice compared to WT mice. N=5. Scale bar=100μm. (**C-E**) Adipocyte EGFR-/- mice and WT mice had similar body weight gain (n=15) (**C**), fasting blood glucose (n=15) (**D**), and HbA1c (N=15 and 17) (**E**). (**F and G**) Adipocyte EGFR-/- mice and WT mice had similar glucose tolerance test (n=6 and 8) (**F**) and insulin tolerance test (n=6) (**G**).

Data are means ± SEM, analyzed using 2 tailed Student's t test for **A** and **E;** 2-way ANOVA followed by Tukey's post hoc test for **C**, **D**, and **G;** and 2 tailed Student's t test and 2-way ANOVA followed by Tukey's post hoc test for **F**.

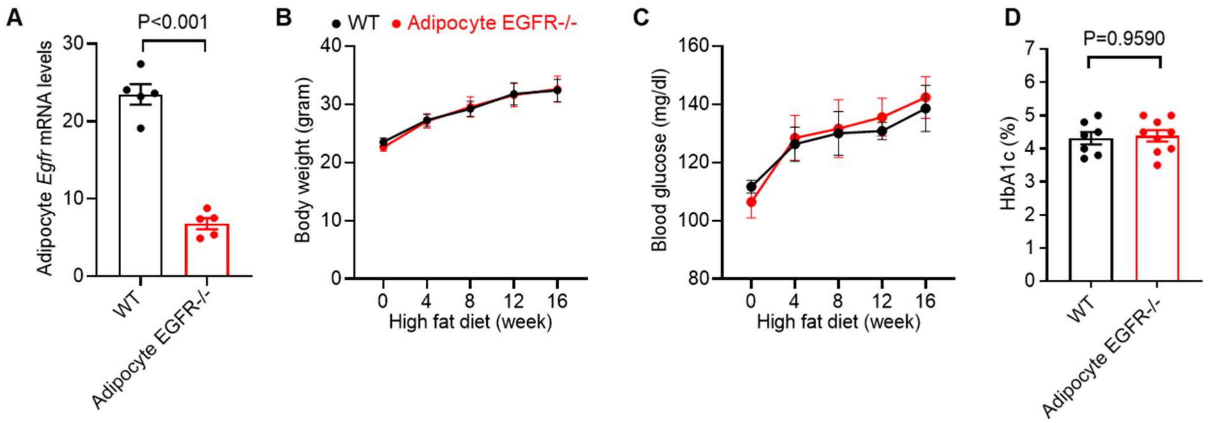

Supplementary Figure 5. Selective deletion of EGFR in adipocytes in female mice had no effect on insulin resistance in diet induced obesity. Female Adipocyte EGFR-/- (Adipoq-Cre; $EGFR^{f/f}$) mice and WT ($EGFR^{f/f}$) mice were fed with the HFD for 16 weeks. (**A**) Inguinal EGFR mRNA expression was markedly decreased in adipocyte EGFR-/- mice compared to WT mice. N=5. (**B**-**D**) Female adipocyte EGFR-/- mice and WT mice had similar body weight gain (**B**), fasting blood glucose (**C**), and HbA1c (**D**). N=7 and 9.

Data are means ± SEM, analyzed using 2 tailed Student's t test for **A** and **D,** 2-way ANOVA followed by Tukey's post hoc test for **B and C.**

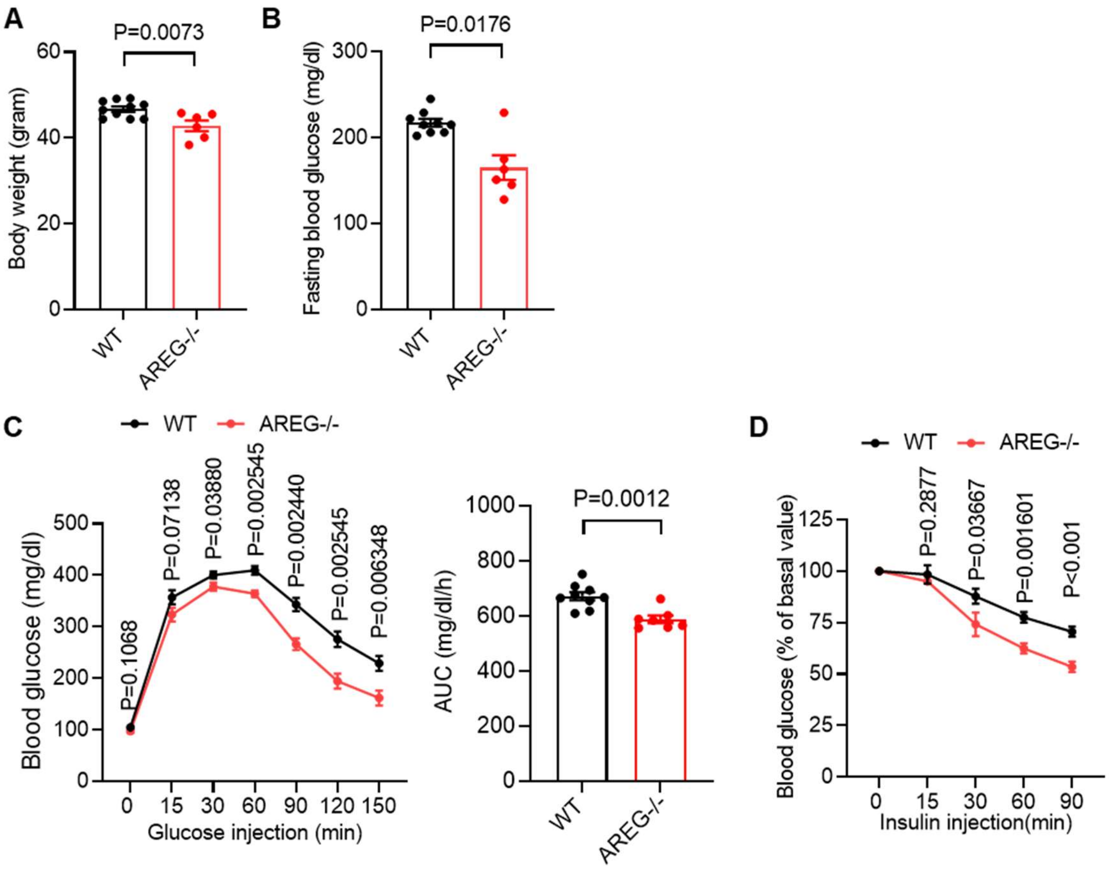

Supplementary Figure 6. AREG deletion protected against insulin resistance in response to a high fat diet. WT and global AREG-/- mice were fed with the HFD for 12 weeks. (**A** and **B**) Global AREG-/- mice had lower body weight (**A**) and lower fasting blood glucose (**B**). N=6 and 10. (**C** and **D**) Global AREG-/- mice had improved glucose tolerance (n=7 and 9) (**C**) and insulin tolerance (n=6 and 9) (**D**).

Data are means ± SEM, analyzed using 2 tailed Student's t test for **A and B**; 2 tailed Student's t test and 2-way ANOVA followed by Tukey's post hoc test for **C;** and 2-way ANOVA followed by Tukey's post hoc test for **D.**

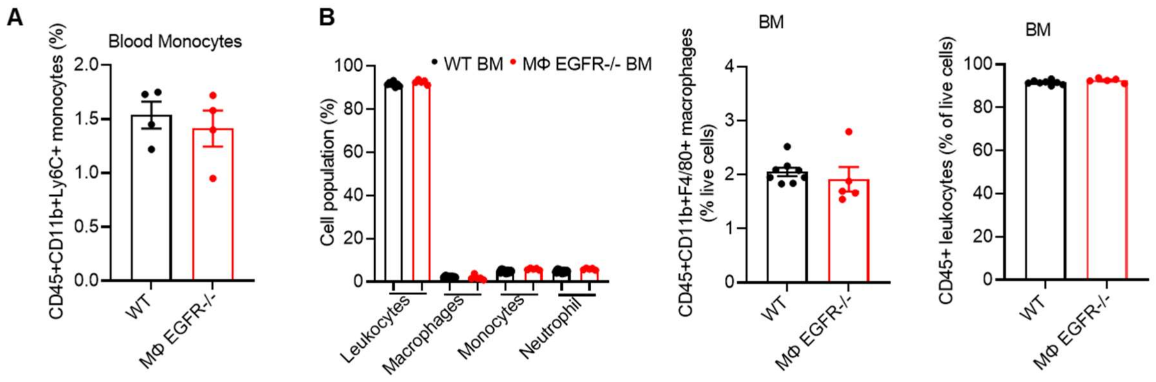

Supplementary Figure 7. Myeloid EGFR deletion had no effect on blood monocyte counts and bone marrow leukocyte composition. Control WT and MΦ EGFR-/- mice were used. (**A)** Flow cytometry determined no differences in the monocyte (CD45+; CD11b+; Ly6C+) population from peripheral blood isolated from WT and MΦ EGFR-/- mice. N=4. (**B)** Flow cytometry also confirmed no significant changes in leukocyte (CD45+), macrophage (CD45+; CD11b+; F4/80+), monocyte (CD45+; CD11b+; F4/80dim; Ly6C+), and neutrophil (CD45+; CD11b+; Ly6G+) bone marrow cell populations relative to total alive bone marrows cells. N=5 and 8.

Data are means ± SEM, analyzed using 2 tailed Student's t test.

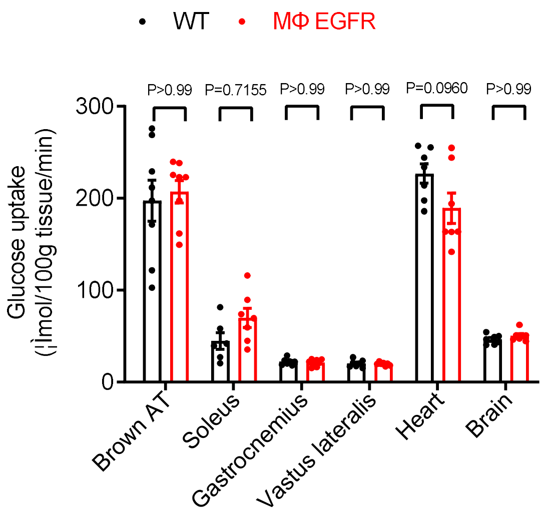

Supplementary Figure 8. Myeloid EGFR deletion had no effect on glucose uptake in any tissues except for white adipose tissues in diet-induced obesity. WT and myeloid EGFR-/- mice were on the HFD for 12 weeks, and hyperinsulinemic-euglycemic clamps were performed. Glucose uptake, a marker of insulin resistance, was comparable in brown AT, skeletal muscles (soleus, gastrocnemius, and vastus lateralis), and heart and brain.

Data are means ± SEM, n=7 and 8, analyzed using 2 tailed Student's t test.

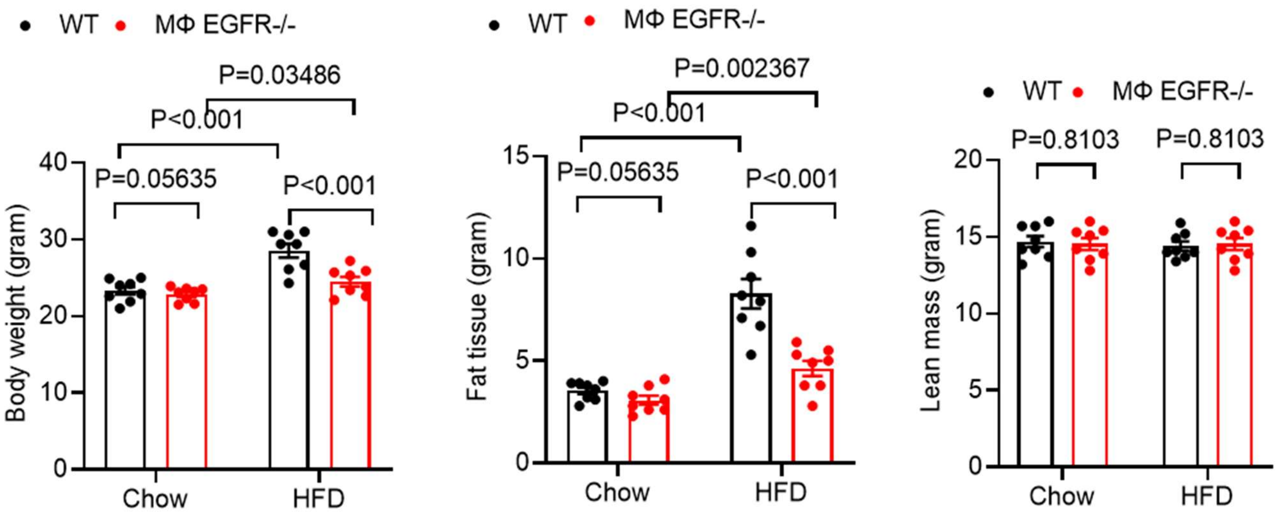

Supplementary Figure 9. High fat diet led to reduced fat mass increases in myeloid EGFR-/- mice. Body composition was determined by NMR in MΦ EGFR-/- mice and littermate WT mice with chow food and with HFD for 4 weeks. Body weight, fat tissue mass and lean mass were comparable between WT and MΦ EGFR-/- mice with chow food diet. HFD for 4 weeks led to less increased body weight and fat tissue mass in MΦ EGFR-/- mice compared to WT mice. N=8.

Data are means ± SEM, analyzed using 2-way ANOVA followed by Bonferroni's post hoc test.

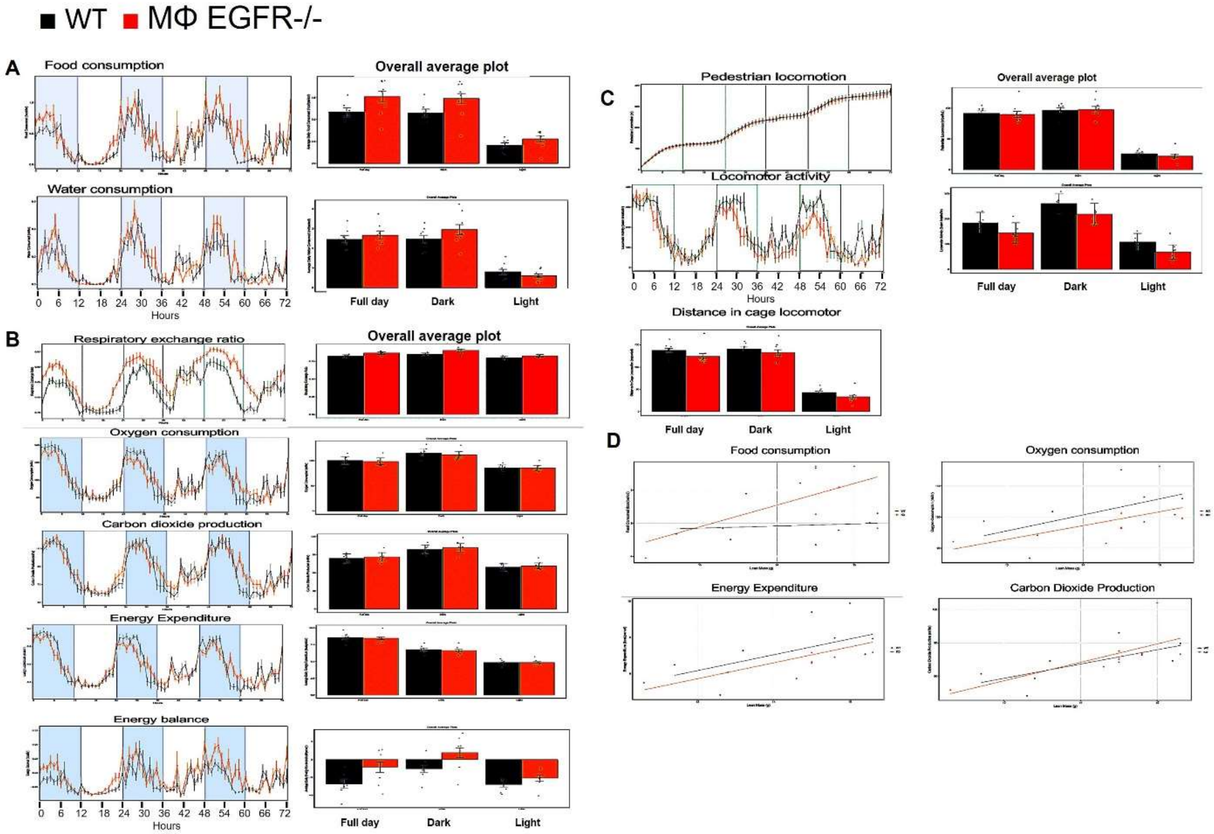

Supplementary Figure 10. WT and myeloid EGFR-/- mice had similar metabolic parameters on chow food diet. We assessed energy balance by indirect calorimetry experiment with Promethion Systems in WT and MΦ EGFR-/- mice fed chow food. (**A**-**C)** No differences were observed in food ingestion and circadian rhythm of feeding (**A**), respiratory exchange ratio, oxygen consumption, carbon dioxide production, energy expenditure, energy balance (**B**), ambulatory/pedestrian locomotion, locomotor activity, and distance in cage locomotor between WT mice and MΦ EGFR-/- mice (**C**). Shaded areas indicate the dark photoperiod. Data are presented by mean ± SEM; n=not significant, by 2-way ANOVA followed by Bonferroni's post hoc test, n=8 in each group. **(D)** Regression plot of food consumption, oxygen consumption, energy expenditure, and carbon dioxide production vs. lean mass. N=8, ANCOVA was used.

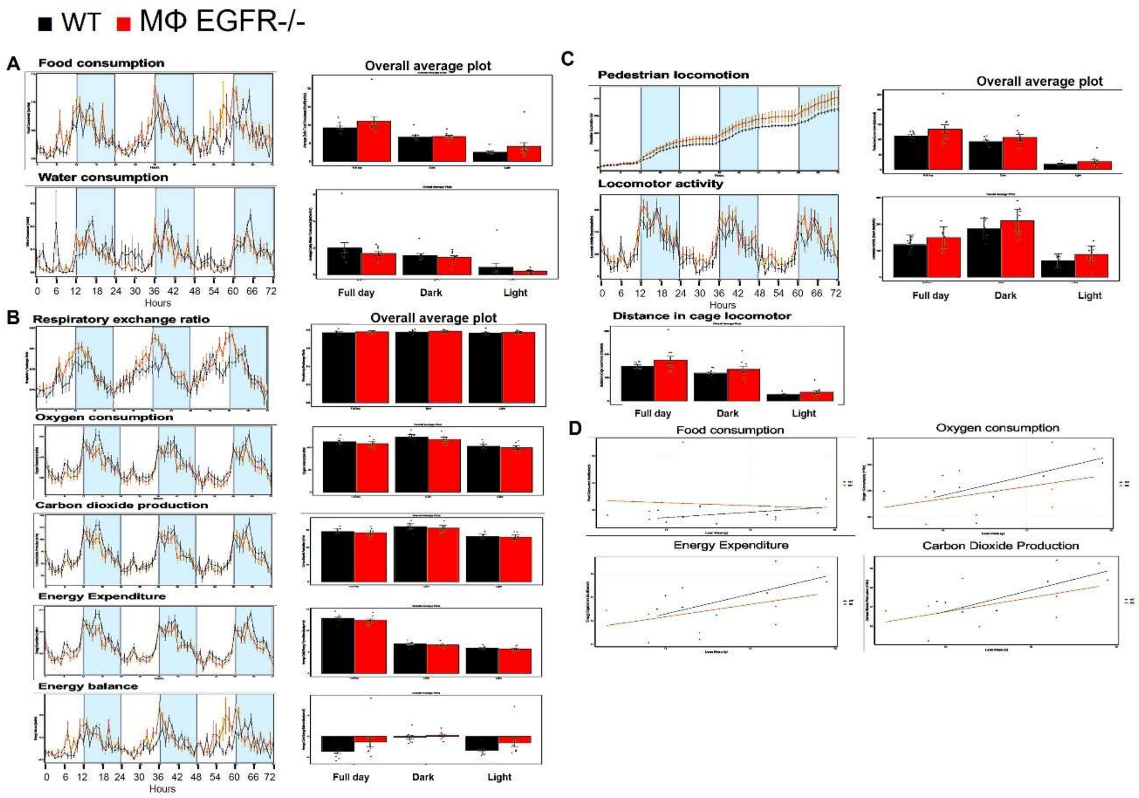

Supplementary Figure 11. WT and myeloid EGFR-/- mice had similar metabolic parameters after 4 weeks on the high fat diet. We assessed energy balance by indirect calorimetry experiment with Promethion Systems in WT and MΦ EGFR-/- mice fed HFD for 4 weeks. (**A**-**C)** No differences were observed in food ingestion and circadian rhythm (**A**), respiratory exchange ratio, oxygen consumption, carbon dioxide production, energy expenditure or energy balance (**B**) between WT mice and MΦ EGFR-/- mice although there was trend for increased ambulatory/pedestrian locomotion, locomotor activity, and distance in cage locomotor in the MΦ EGFR-/- mice (**C**). Shaded areas indicate the dark photoperiod. Data are presented by mean ± SEM; by 2-way ANOVA followed by Bonferroni's post hoc test, n=8 in each group. (**D**) Regression plot of food consumption, oxygen consumption, energy expenditure, and carbon dioxide production vs. lean mass. N=8, ANCOVA was used.

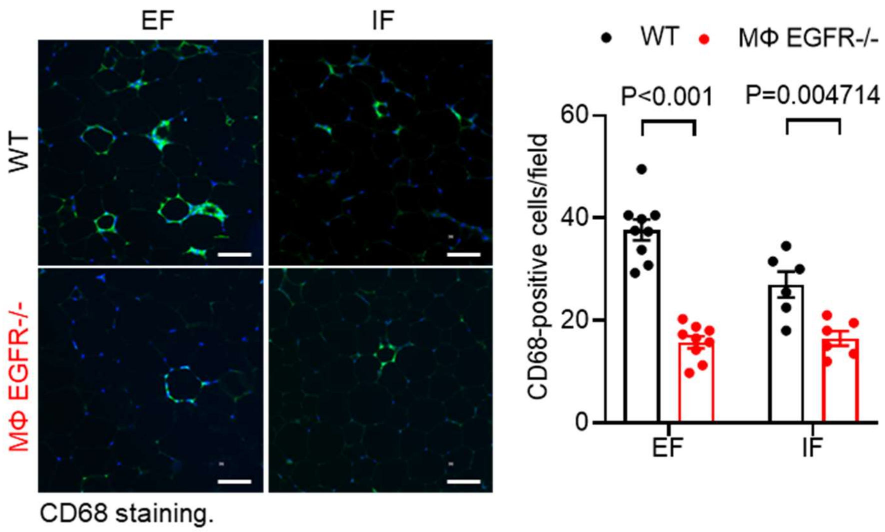

Supplementary Figure 12. Mice with myeloid EGFR deletion had fewer ATMs in diet-induced obesity. Male WT and myeloid EGFR-/- mice were fed the HFD for 12 weeks. CD68 immunofluorescent staining showed that myeloid EGFR-/- mice had decreased ATMs in both EF and IF. N=6. Scale bar=100μm.

Data are means ± SEM, analyzed using 2-way ANOVA followed by Bonferroni's post hoc test.

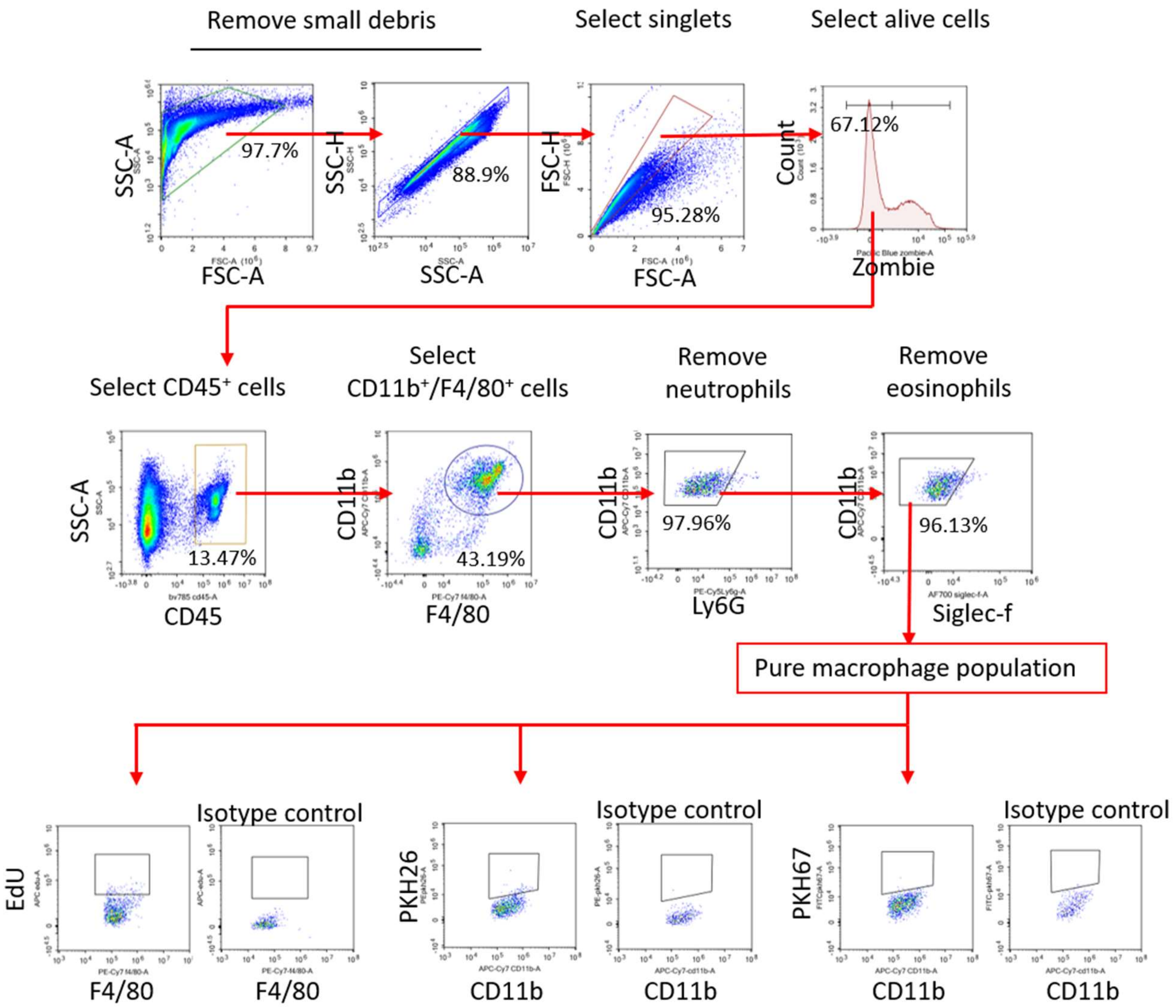

Supplementary Figure 13. Gating strategy of Figure 7B for epididymal fat tissue ATM population, monocyte recruitment, and ATM proliferation analysis.

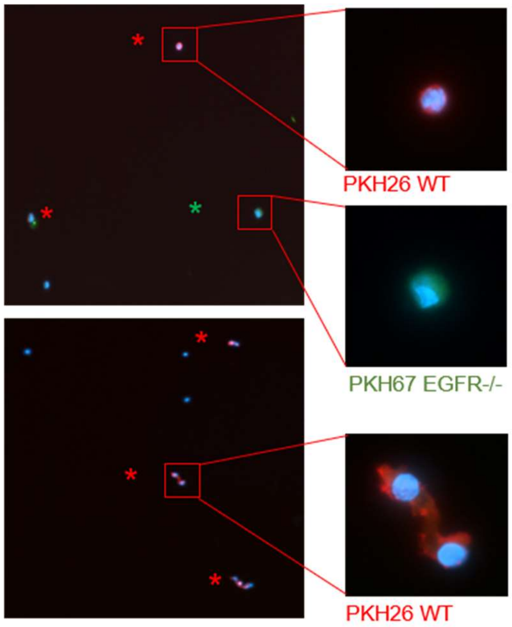

Supplementary Figure 14. Bone marrow derived monocyte (BMDMs) with EGFR deletion had impaired recruitment in WT mice in diet-induced obesity. WT BMDMs were labeled with the monocyte tracking dye PKH26 (red) and EGFR-/- BMDMs with PKH67 (green) and injected a mixture containing equal amounts of labeled WT and EGFR-/- BMDMs into WT recipients fed the HFD for 6 weeks. More PKH26-positive WT BMDMs were also observed in cell suspension smears of EF from WT recipients (representative of 3 independent experiments).

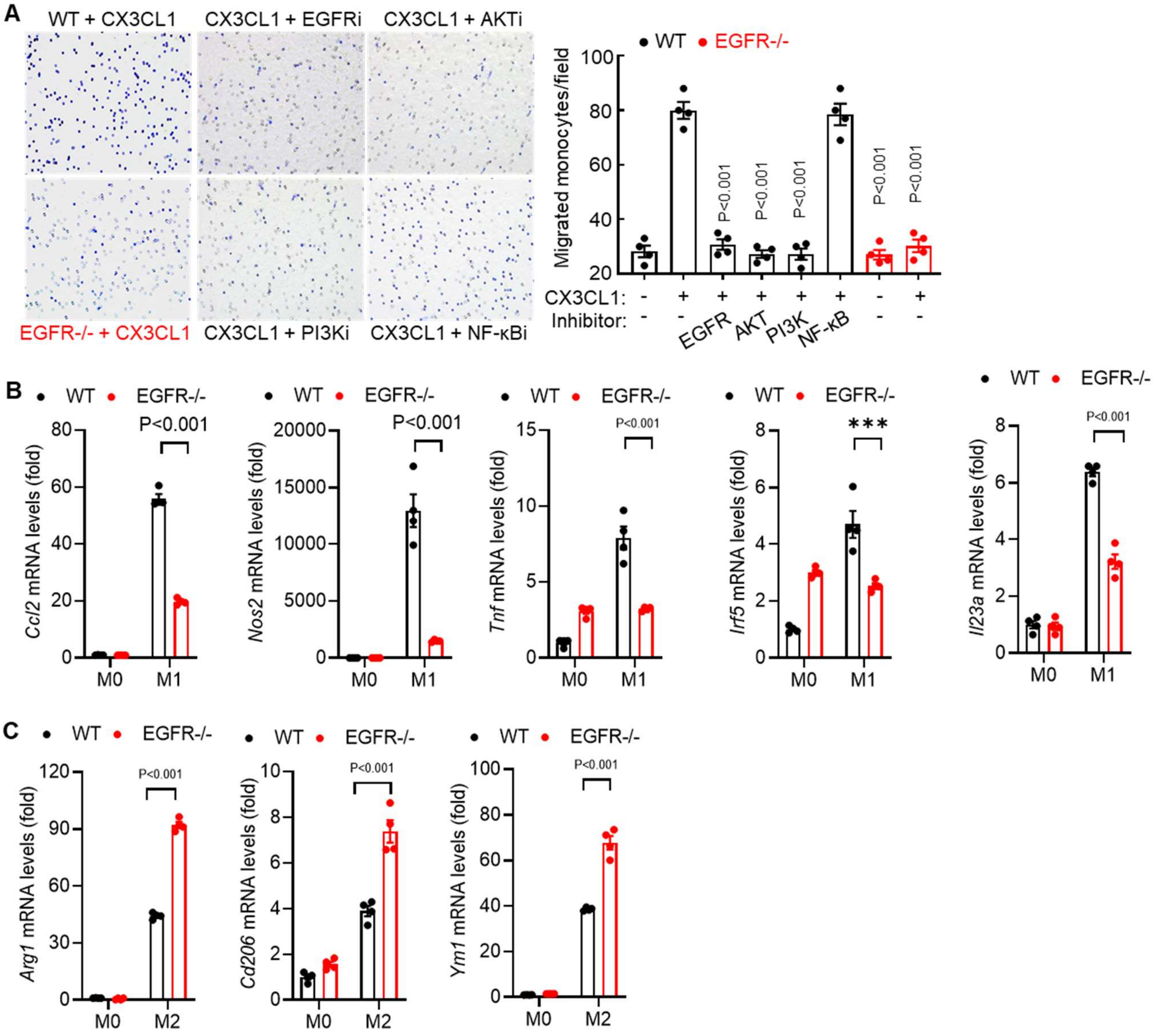

Supplementary Figure 15. EGFR signaling regulated BMDM migration and polarization. BMDMs were isolated from WT and MΦ EGFR-/- mice. (**A**) Chemoattractant CX3CR1-induced migration in WT BMDMs was blocked by an EGFR inhibitor (erlotinib), an AKT inhibitor (MK-2206), a PI3K inhibitor (BEZ235) or EGFR deletion. N=4. (**B** and **C**) EGFR-/- BMDMs had lower proinflammatory M1/Th1 cytokines/chemokines with LPS/IFN-γ-mediated M1 polarization, including *Ccl2*, *Nos2*, *Tnf*, and *Il23a* (**B**) but higher anti-inflammatory cytokines with IL-4/IL-13-mediated M2 polarization, including *Arg1*, *Cd206*, and *Ym1* (**C**). N = 4 in each group.

Data are means ± SEM, analyzed using 2-way ANOVA followed by Bonferroni's post hoc test for all.

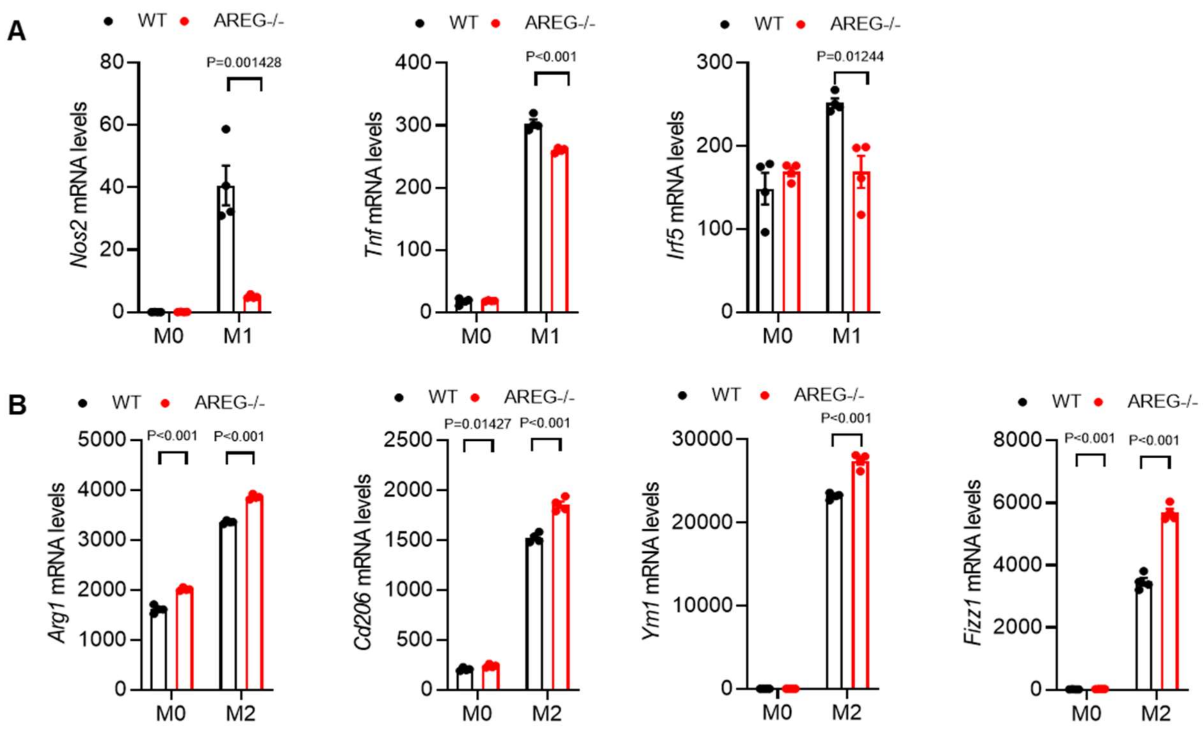

Supplementary Figure 16. AREG regulated BMDM polarization. BMDMs were isolated from WT and global AREG-/- mice. (**A** and **B**) AREG-/- BMDMs had lower proinflammatory M1/Th1 cytokines/chemokines with LPS/IFN-γ-mediated M1 polarization, including *Nos2*, *Tnf*, and *Irf5* (**A**) but higher anti-inflammatory cytokines with IL-4/IL-13-mediated M2 polarization, including *Arg1*, *Cd206*, *Fizz1*, and *Ym1* (**D**). N = 4 in each group.

Data are means ± SEM, analyzed using 2-way ANOVA followed by Bonferroni's post hoc test for all.

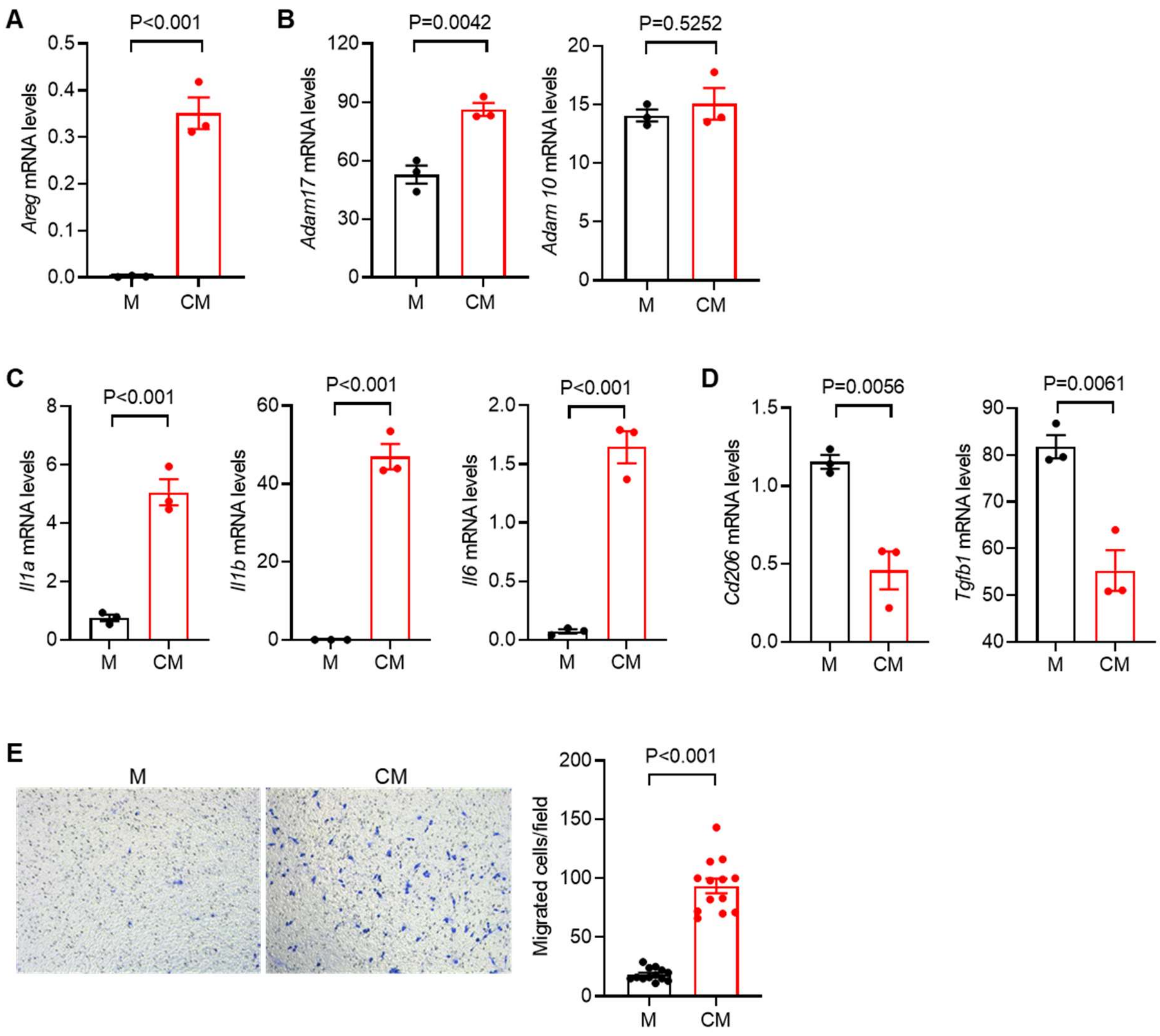

Supplementary Figure 17. Adipocytes regulated macrophage properties. Apoptotic 3T3-L1 cell differentiated adipocytes and mouse macrophage-like RAW264.7 cells were used. **(A-D)** Compared to serum-free medium (M), conditional serum-free medium (CM) from apoptotic adipocytes stimulated RAW264.7 cell mRNA expression of *Areg* (**A**), *Adam17* (**B**), proinflammatory cytokines including *Il1a*, *Il1b*, and *Il6* (**C**), and inhibited antiinflammatory *Cd206* and *Tgfb* (**D**). N=3. (**E**) Coculture with apoptotic adipocytes led to increased RAW264.7 cell migration. N=13.

Data are means ± SEM, analyzed using 2 tailed Student's t test.

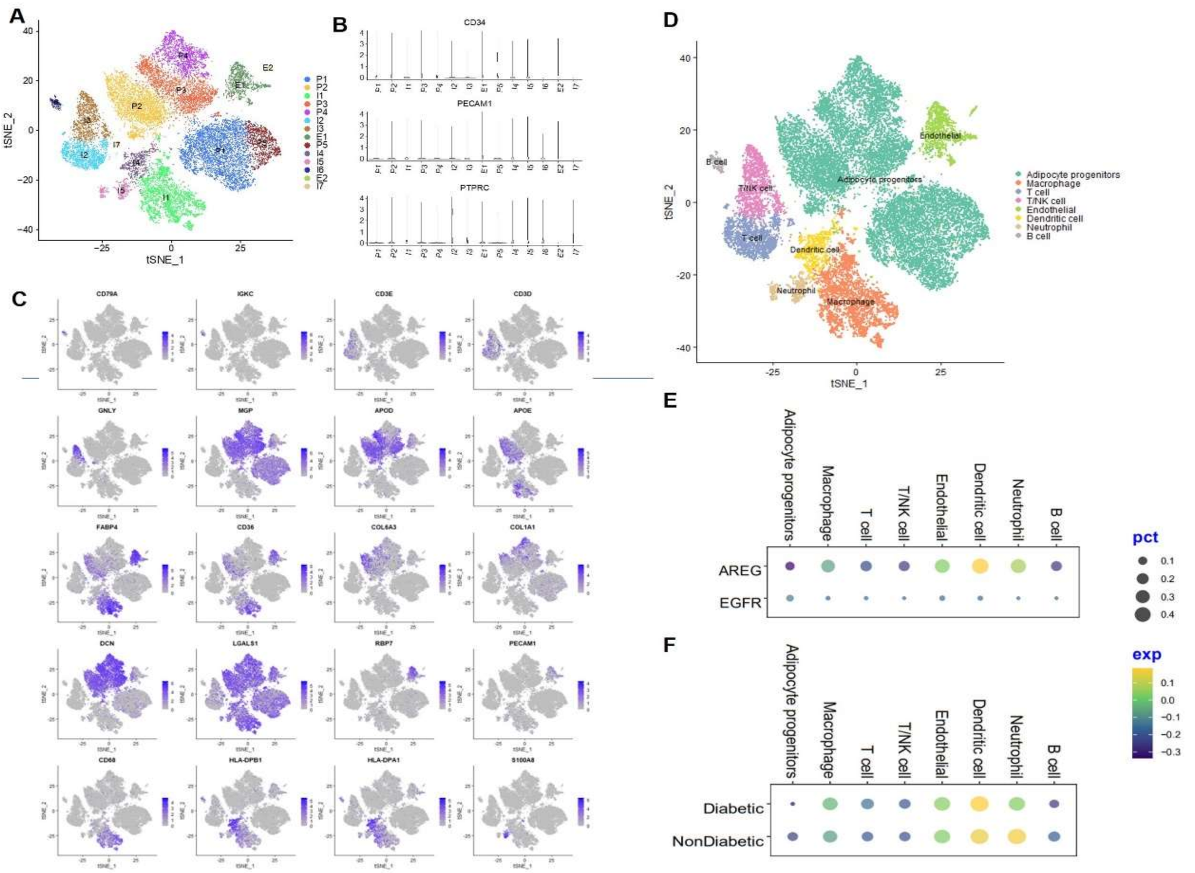

Supplementary Figure 18. *AREG* mRNA levels were increased in human adipose tissue macrophages and dendritic cells in individuals with type 2 diabetes. **A**. Identified cell populations in the non-adipocyte fraction of adipose tissue from database (GSE129363)[27]. Fourteen clusters were identified from 26,350 cells derived from SVF (stromal vascular fraction) of 25 adipose samples, including progenitors (P1–P5), immune cells (I1–I7) and endothelial cells (E1–E2). **B**. SVF-derived progenitor clusters. Violin plots of log-transformed expression density of *CD34*, *CD31/PECAM1* and *CD45/PTPRC* across all SVF clusters from 25 samples. The width of the violin plot indicates the number of cells expressing the particular gene. **C**. Cell type annotation indicated cell populations classified as immune cells. **D**. Six clusters of immune cells were identified, macrophages, T cells, T/NK cells, dendritic cells, neutrophils, and B cells. **E**. *AREG* transcripts were evident in macrophages, dendritic cells, neutrophils, and endothelial cells. **F**. Macrophage and dendritic cell *AREG* transcripts were higher in diabetic adipose tissue than in non-diabetic adipose tissue.

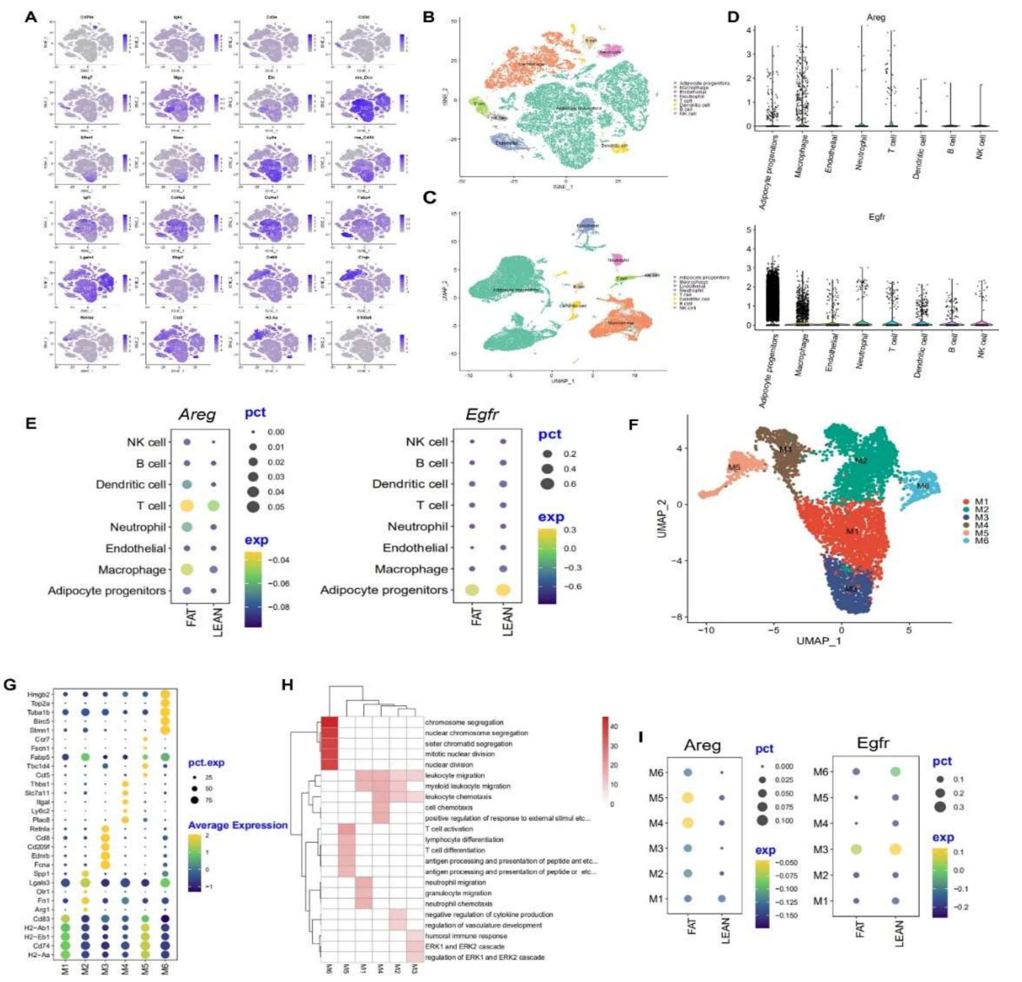

Supplementary Figure 19. *Areg* mRNA levels were increased in adipose tissue macrophages in high fat diet induced obese mice. **A**. Cell type annotation for cell populations from epididymal fat tissue stromal vascular fraction (SVF) of lean and fat mice from GSE16187 database[28]. **B**. Clustering results indicated six clusters of immune cell populations including macrophages, T cells, NK cells, dendritic cells, neutrophils, and B cells. **C**. Uniform manifold approximation and projection (**UMAP**) plot for unsupervised clustering of the single cells identified a total of 8 cell populations. **D**. Both *Areg* and *Egfr* transcripts were mainly expressed in adipocyte progenitors and macrophages. **E**. *Areg* transcripts were increased in macrophages, dendritic cells, neutrophils, and T cells in SVF from obese mice. There were no differences in *Egfr transcripts* between lean and obese mice. **F**. UMAP plot for unsupervised clustering of the single cells identified a total of 6 macrophage populations (M1-M6). G. Bubble map shows the top genes in all macrophage subgroups. **H**. Gene ontology analysis of the M1 to M6 macrophage subgroups. **I**. *Areg* transcripts were most increased in M4 and M5 subgroups in obese mice. GO analysis revealed M4 and M5 subgroups are involved in leukocyte migration and T cell activation. There were no differences in *Egfr* transcripts in macrophage subgroups between lean mice and obese mice.
