# Supplementary material for: EGFR-mediated activation of adipose tissue macrophages promotes obesity and insulin resistance
Source: Nat Commun. 2022 Aug 10;13:4684. doi: 10.1038/s41467-022-32348-3 (PMC9365849; doi:10.1038/s41467-022-32348-3)
Supplement: Supplementary file 2 — Reporting Summary [file 41467_2022_32348_MOESM2_ESM.pdf]

nature portfolio

Corresponding author(s): NCOMMS-21-29467C

Last updated by author(s): 2022 06 15

Reporting Summary

Nature Portfolio wishes to improve the reproducibility of the work that we publish. This form provides structure for consistency and transparency in reporting. For further information on Nature Portfolio policies, see our Editorial Policies and the Editorial Policy Checklist.

Statistics

For all statistical analyses, confirm that the following items are present in the figure legend, table legend, main text, or Methods section.

n/a	Confirmed	
☐	☒	The exact sample size (*n*) for each experimental group/condition, given as a discrete number and unit of measurement
☐	☒	A statement on whether measurements were taken from distinct samples or whether the same sample was measured repeatedly
☐	☒	The statistical test(s) used AND whether they are one- or two-sided<br>*Only common tests should be described solely by name; describe more complex techniques in the Methods section.*
☒	☐	A description of all covariates tested
☒	☐	A description of any assumptions or corrections, such as tests of normality and adjustment for multiple comparisons
☐	☒	A full description of the statistical parameters including central tendency (e.g. means) or other basic estimates (e.g. regression coefficient) AND variation (e.g. standard deviation) or associated estimates of uncertainty (e.g. confidence intervals)
☐	☒	For null hypothesis testing, the test statistic (e.g. *F*, *t*, *r*) with confidence intervals, effect sizes, degrees of freedom and *P* value noted<br>*Give P values as exact values whenever suitable.*
☒	☐	For Bayesian analysis, information on the choice of priors and Markov chain Monte Carlo settings
☒	☐	For hierarchical and complex designs, identification of the appropriate level for tests and full reporting of outcomes
☒	☐	Estimates of effect sizes (e.g. Cohen's *d*, Pearson's *r*), indicating how they were calculated

Our web collection on statistics for biologists contains articles on many of the points above.

Software and code

Policy information about availability of computer code

Data collection	1.Nikon TE300 fluorescence microscope and spot-cam digital camera (Diagnostic Instruments) and Leica DMil microscope: Images data collection.<br>2. TaqMan real-time PCR (7900HT, Applied Biosystems): Quantitative RT-PCR data collection;<br>3. NovoCyte® Quanteon Flow Cytometer: Flow cytometry data collection;<br>4. B-Glucose Analyzer (HemoCue, Lake Forest, CA): Blood glucose data collection;<br>5. FluorChem™ system (Bio-Techne, US): Western blot and DNA gels data collection;<br>6. Indirect calorimetry (Promethion,Sable Systems, Las Vegas, NV): Energy expenditure data collection;<br>7. MetaScreen and MacroInterpreter (Sable Systems): Ambulatory activity data acquisition and processing coordination;<br>8. NMR (Bruker Minispec): Body composition data collection.
Data analysis	1. Iimage J software (NIH, Bethesda, MD): Images data analysis;<br>2. QuantStudio 7 Pro Real-Time PCR Systems Software:Quantitative RT-PCR data analysis;<br>3. NoveExpress® Software: Flow cytometry data analysis;<br>4. Seurat package (version 3.2.0) (Satija et al.2019):Unsupervised clustering ,dimensionality reductionand and differential gene expression analyses;<br>5. The Weir equation: EE (kcal/hr)= 60*(0.003941 *VO2(ml/min) +0.001106*VCO2(ml/min): Energy expenditure calculation;<br>6. All statistical analyses were performed with Graph Pad Prism9 (GraphpadSoftware® Inc., La Jolla, CA, US).

For manuscripts utilizing custom algorithms or software that are central to the research but not yet described in published literature, software must be made available to editors and reviewers. We strongly encourage code deposition in a community repository (e.g. GitHub). See the Nature Portfolio guidelines for submitting code & software for further information.

Data

Policy information about availability of data

All manuscripts must include a data availability statement. This statement should provide the following information, where applicable:

- Accession codes, unique identifiers, or web links for publicly available datasets
- A description of any restrictions on data availability
- For clinical datasets or third party data, please ensure that the statement adheres to our policy

All the data supporting the findings of this study are available within the article and its Supplementary Information files. A reporting summary for this article is available as Supplementary Information. Source data are provided with this paper.

Human research participants

Policy information about studies involving human research participants and Sex and Gender in Research.

Reporting on sex and gender	Not applied
Population characteristics	Not applied
Recruitment	Not applied
Ethics oversight	Not applied

Note that full information on the approval of the study protocol must also be provided in the manuscript.

Field-specific reporting

Please select the one below that is the best fit for your research. If you are not sure, read the appropriate sections before making your selection.

[x] Life sciences [ ] Behavioural & social sciences [ ] Ecological, evolutionary & environmental sciences

For a reference copy of the document with all sections, see nature.com/documents/nr-reporting-summary-flat.pdf

Life sciences study design

All studies must disclose on these points even when the disclosure is negative.

Sample size	The samples sizes were determined according to our preliminary data. We used 8-10 mice in each group as our preliminary data indicated that a >30% difference could be achieved. For in vitro study, our preliminary data showed S.D. was <15% and difference was at least >100%, and N is 3-4 was chosen.
Data exclusions	We did not exclude data for both in vivo and in vitro experiments.
Replication	After our preliminary proof of concept experiments, we performed our studies and all attempts at replication were successful.
Randomization	We allocated animals randomly for our experiments.
Blinding	The investigators were blinded to group allocation, data collection and analysis.

Reporting for specific materials, systems and methods

We require information from authors about some types of materials, experimental systems and methods used in many studies. Here, indicate whether each material, system or method listed is relevant to your study. If you are not sure if a list item applies to your research, read the appropriate section before selecting a response.

Materials & experimental systems

n/a	Involved in the study
	☒ Antibodies
	☒ Eukaryotic cell lines
☒	Palaeontology and archaeology
	☒ Animals and other organisms
☒	Clinical data
☒	Dual use research of concern

Methods

n/a	Involved in the study
☒	ChIP-seq
	☒ Flow cytometry
☒	MRI-based neuroimaging

Antibodies

Antibodies used	All antibody information is included in Supplementary Table S2.
Validation	All antibodies were purchased from vendors such as Sigma, abcam, CST, Biolegend, and the selectivity of the antibody is validated by manufacturers such as with knockdown cell cells.

Eukaryotic cell lines

Policy information about cell lines and Sex and Gender in Research

Cell line source(s)	Both RAW264.6 (ATCC TIB-71) and 3T3-L1 (CL-173™) cells were purchased from ATCC.
Authentication	Both cell lines were authenticated by morphological analysis, as compared to the ATCC documentation.
Mycoplasma contamination	The cell lines were tested negative for mycoplasma contamination.
Commonly misidentified lines (See ICLAC register)	not available.

Animals and other research organisms

Policy information about studies involving animals; ARRIVE guidelines recommended for reporting animal research, and Sex and Gender in Research

Laboratory animals	C57BL/6, 8-10 weeks mice were used at the beginning of the experiments.
Wild animals	n/a
Reporting on sex	Both male and female were used for the experiments. However, male and female were used in different groups.
Field-collected samples	n/a
Ethics oversight	All animal experiments were performed in accordance with the guidelines and approval of the Institutional Animal Care and Use Committee of Vanderbilt University.

Note that full information on the approval of the study protocol must also be provided in the manuscript.

Flow Cytometry

Plots

Confirm that:

- ☒ The axis labels state the marker and fluorochrome used (e.g. CD4-FITC).
- ☒ The axis scales are clearly visible. Include numbers along axes only for bottom left plot of group (a 'group' is an analysis of identical markers).
- ☒ All plots are contour plots with outliers or pseudocolor plots.
- ☒ A numerical value for number of cells or percentage (with statistics) is provided.

Methodology

Sample preparation	Epididymal fat tissue cell suspension was prepared using DNAse1 (56U/ml, Bio-Rad#7326828) and collagenase D (4mg/ml, Roche#11088882001), and then suspended in 100 µl of PBS containing 1% BSA and incubated with 0.5 µl Fc block (BD Pharmingen, purified Rat anti-Mouse CD16/CD32. Anti-CD45, anti-CD11b, and anti-F4/80 were used to identify ATMs Appropriate isotype controls were included for each sample.

Instrument	NovoCyte Quanteon Flow Cytometer Systems.
Software	NoveExpress Software
Cell population abundance	After removal of death cells, neutrophils and macrophages were evaluated.
Gating strategy	Gating strategy is provided in Supplementary Figure S13.

☒ Tick this box to confirm that a figure exemplifying the gating strategy is provided in the Supplementary Information.
